# Azaarenes: 13 Rings in a Row by Cyclopentannulation

**DOI:** 10.1002/anie.202214031

**Published:** 2022-12-22

**Authors:** Steffen Maier, Nikolai Hippchen, Fabian Jester, Marcus Dodds, Michel Weber, Leon Skarjan, Frank Rominger, Jan Freudenberg, Uwe H. F. Bunz

**Affiliations:** ^1^ Organisch-Chemisches Institut Ruprecht-Karls-Universität Heidelberg Im Neuenheimer Feld 270 69120 Heidelberg Germany; ^2^ Centre for Advanced Materials Ruprecht-Karls-Universität Heidelberg Im Neuenheimer Feld 225 69120 Heidelberg Germany

**Keywords:** Aromaticity, Azaacenes, Azaarenes, Cyclopentannulation, Molecular Nanocarbons

## Abstract

Cyclopentannulation was explored as a strategy to access large, stable azaarenes. Buchwald–Hartwig coupling of previously reported di‐ and tetrabrominated cyclopentannulated *N*,*N*′‐dihydrotetraazapentacenes furnished stable azaarenes with up to 13 six‐membered rings in a row and a length of 3.1 nm. Their optoelectronic and semi‐conducting properties as well as their aromaticity were investigated.

## Graphical abstract

Large, stable cyclopentannulated azaarenes, consisting of up to 13 six‐membered rings in a row, were prepared. Cyclopentannulation complements benzannulation strategies for the preparation of the higher (aza)arenes.

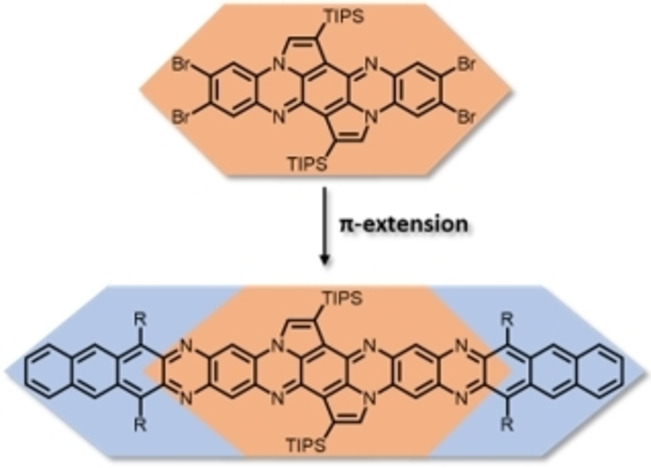

Soluble, one‐dimensional polycyclic aromatic hydrocarbons^[1]^ (molecular nanocarbons^[2]^) are models of rigid nanowires.^[3]^ Being monodisperse, they are defined, purified and completely characterized species, while their ladder polymer relatives^[4]^ are invariably polydisperse and may contain defects.^[5]^ Oligomers of increasing length are a synthetic challenge but of interest as charge carrier materials and allow to study electronic communication along π‐systems in respect to conjugation lengths,^[6]^ and aromaticity effects.^[7]^

The simplest one‐dimensional molecular nanocarbons are the larger acenes.^[8]^ These are limited in length due to their instability. Thus far the largest homologues prepared are dodecacene^[9]^ by on‐surface and nonacene^[10]^ by solution‐phase and solid‐state^[11]^ syntheses. To lower their di‐ and oligoradical character,^[12]^ responsible for degradation, several annulation strategies have been developed to stabilize the molecular backbones.[^6d^, ^7b^, ^13^] The number of Clar sextets is increased by combining acene fragments with other small hydrocarbons, such as perylene,[^14^, ^15^] corannulene,^[15]^ pyrene,[^5a^, ^13h^, ^13k^, ^16^] phenanthrene,^[13b]^ *para*‐benzoquinone^[17]^ and its Knoevenagel derivatives (Figure 1, **A**).^[18]^ Synthetic strategies involve condensation reactions, especially for N‐doped nanocarbons such as (**B**),[^23a^, ^29^] Buchwald–Hartwig reactions (**C**),^[15]^ Suzuki couplings and Friedel–Crafts ring closures (**D**)^[28]^ as well as Cava reactions (**A**), cyclo‐reversions of butterfly dimers^[19]^ or removal of thermo‐ or photocleavable groups^[20]^ to generate the intact π‐system in the last synthetic step. Coupling of arene‐type building blocks is achieved either in one‐pot or with protection strategies (Alonso et al.,^[21]^ Baumgarten et al.^[22]^) or by zipping up a long precursor (Mastalerz et al.,[^14c^, ^23^] Chalifoux et al.^[24]^). The current record holder in length is compound **B** with a length of 14.9 nm.^[25]^

**Figure 1:**
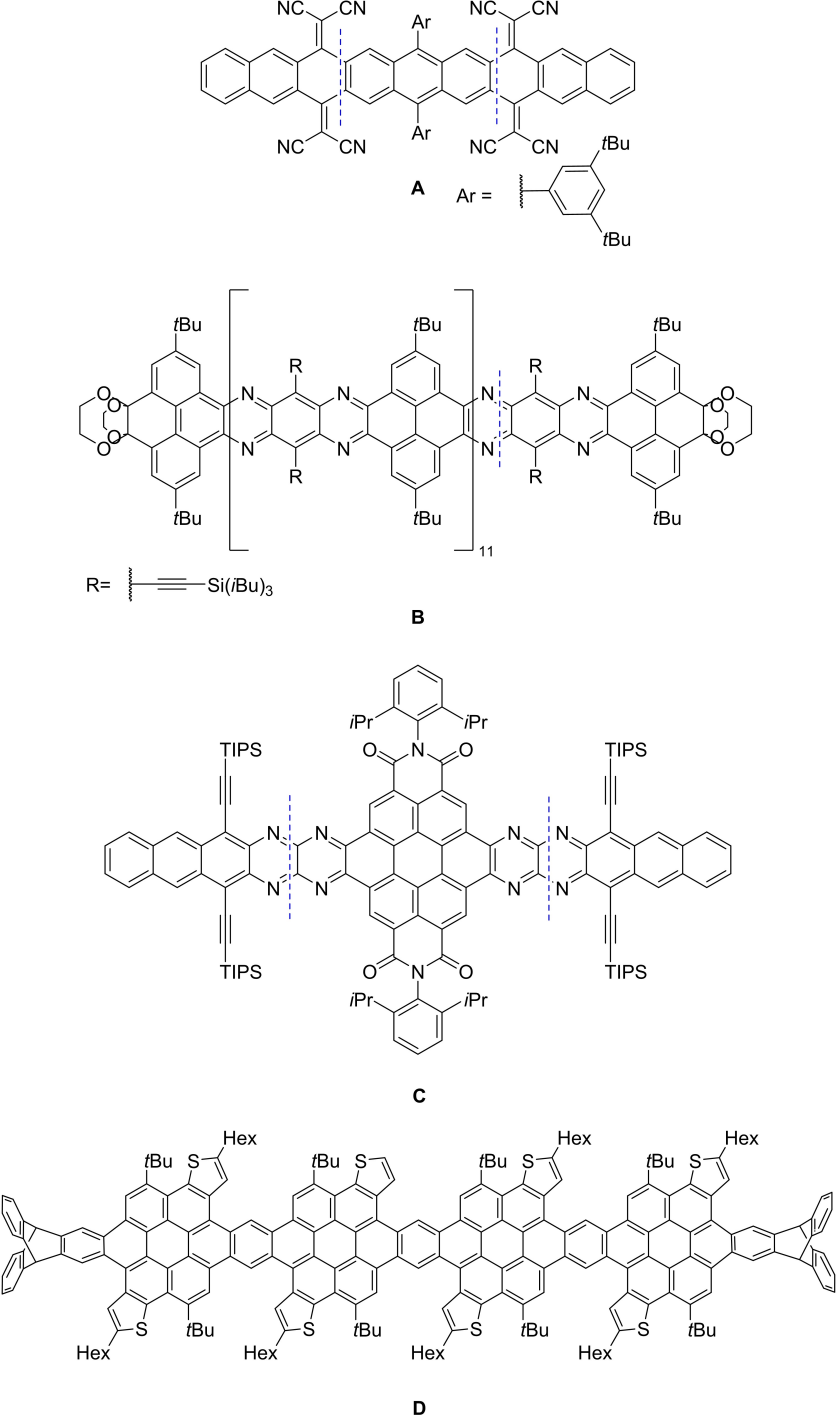
Structures of selected literature known one‐dimensional nanocarbons.[^14c^, ^15^, ^23a^, ^28^]

Most of these examples are based on the annulation of six‐membered rings, whereas cyclopentannulation is underexplored for the synthesis of molecular nanocarbons, although pentacene was previously stabilized in such a fashion.^[26]^

In this contribution, we use our recently reported di‐ and tetrabrominated pentannulated dihydrotetraazapentacene building blocks (**1 a**,**b**)^[27]^ for cross‐coupling reactions with privileged *ortho*‐diamines to obtain soluble molecular N‐doped nanocarbons reaching up to 3.1 nm (calculated by DFT for **3 c**) in length.

Compounds **1 a**,**b** were postfunctionalized via Buchwald–Hartwig^[29]^ couplings with TIPS‐ethynylated diamines^[30]^ (see Supporting Information) furnishing mono‐ (**2 a**–**c**) and bis‐π‐extended (**3 a**–**c**) cyclopentannulated azaarenes along with their corresponding NH‐species (Scheme 1). After column chromatography, the product mixtures were oxidized with manganese(IV) oxide in DCM to yield the oxidized species. The yields ranged between 21 % and 81 %. In contrast to the sparingly soluble starting bromides, the products are well soluble in common organic solvents, testimony to the presence of four or six silyl groups, respectively. Nevertheless, we noticed decreasing solubility with increasing number of rings.

**Scheme 1:**
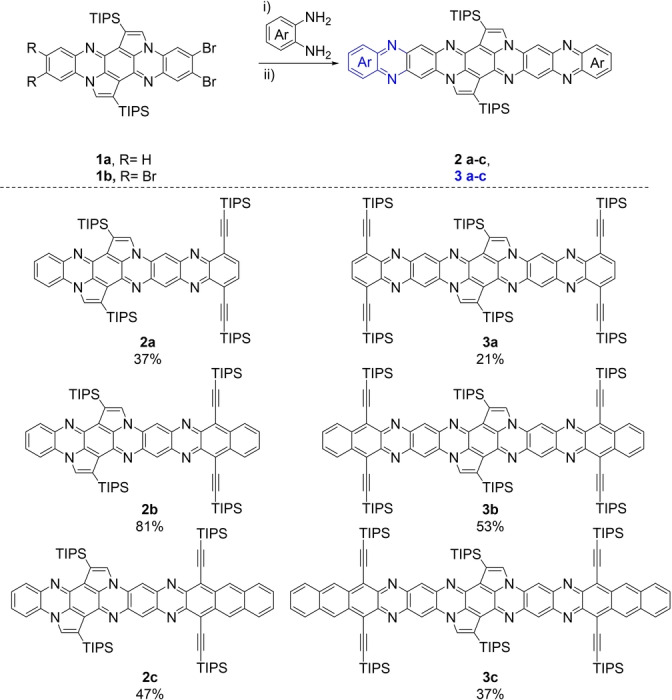
Synthesis of the postfunctionalized cyclopentannulated tetraazapentacenes including yields. Conditions: i) diamine, RuPhos Pd G1, Cs_2_CO_3_, toluene, 140 °C, 15 h; ii) MnO_2_, DCM, rt, 1 h.

Figure 2 displays the absorption spectra of the doubly post‐functionalized but non‐emissive compounds **3 a**–**c** (see Supporting Information, Figure S13, for the spectra of compounds **2 a**–**c**). As expected, the extension of the π‐system results in a red shift of the absorption maximum, increasing number of annulated rings (102 nm for **3 a**, 215 nm for **3 b** and 355 nm for **3 c**) compared to **1 b**. The corresponding mono‐postfunctionalized compounds **2 a**–**c** are blue shifted by 25 nm for the smallest system and up to 32 nm for the largest system with respect to their doubly functionalized counterparts. This indicates only a small electronic interaction between both terminal acene moieties. NICS(1.7)_zz_‐xy‐scans^[31]^ (Figure 3a) as well as anisotropy of the induced current density (AICD‐π‐only)^[32]^ calculations support this interpretation. The central six‐membered ring (ring 7) of **3 c** exhibits NICS(1.7)_zz_ values up to −1 ppm indicating (close to) no aromaticity. In contrast, to the five membered rings are attributed NICS values up to −14 ppm, which is in the same region as the eastern/western rings of the molecule (see Figure 3, rings 1 and 13), a sign of aromaticity. The current density vectors on the AICD‐π‐only isosurface show (diatropic) ring currents over the complete molecule except the two bonds between the central ring and the neighboring six membered rings. In these bonds, scalar field/current density vectors are not observable, indicating the lack of conjugation/aromaticity, in agreement with the NICS calculations (Figure 3c). We calculated the diradical character for **3 a** and **3 c**. Both molecules show a diradical character of 0.

**Figure 2:**
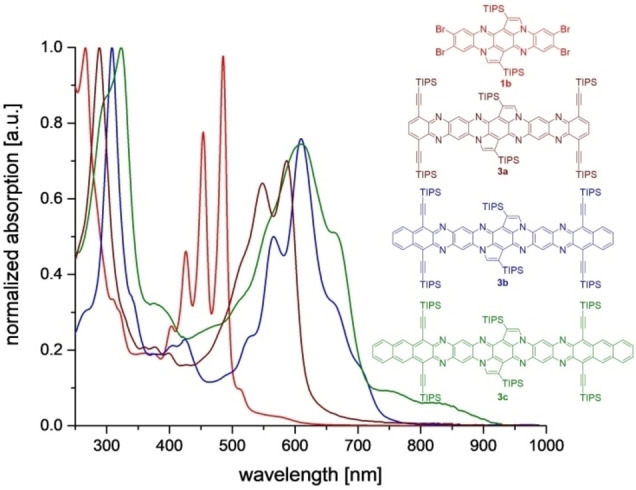
Normalized absorption spectra of the disubstituted derivatives **3 a**, **3 b** and **3 c** and the corresponding starting material **1 b** in DCM.

**Figure 3:**
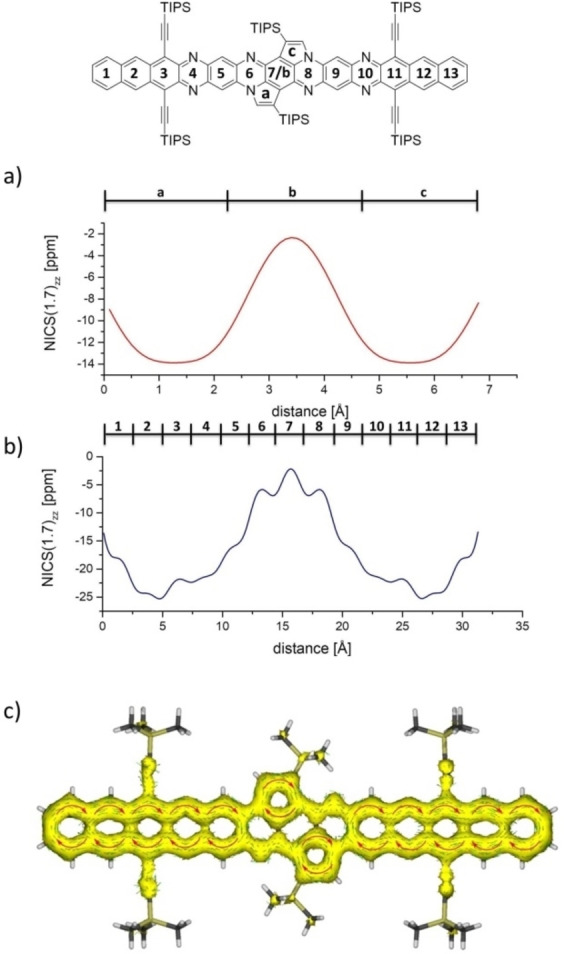
a) NICS(1.7)_zz_ xy scan of **3 c** in y‐direction; b) NICS(1.7)_zz_ xy scan of **3 c** in x‐direction (Gaussian16 B3LYP/def2TZVP, GIAO method); c) AICD‐π‐only plot of **3 c** (Gaussian16 B3LYP/def2TZVP, CSGT method, IOP(10/93=1), optimization limit: 0.01, isovalue: 0.01).

The calculated (DFT, B3LYP/def2‐TZVP) and experimental optoelectronic properties are summarized in Table 1. For the arenes, two reversible reduction events (three for **3 a**) and up to two non‐reversible oxidation events (one for **2 a**, **3 a**, **3 b**, **3 c** and two for **2 b**, **2 c**) were observed in their cyclic voltammograms. Some of the measured values for the electron affinity differ significantly from the calculated ones for the LUMO, probably due to degradation of the compounds on the electrode. TD‐DFT calculations indicate that the absorption maxima correspond to the HOMO–LUMO‐transitions.

**Table 1:**
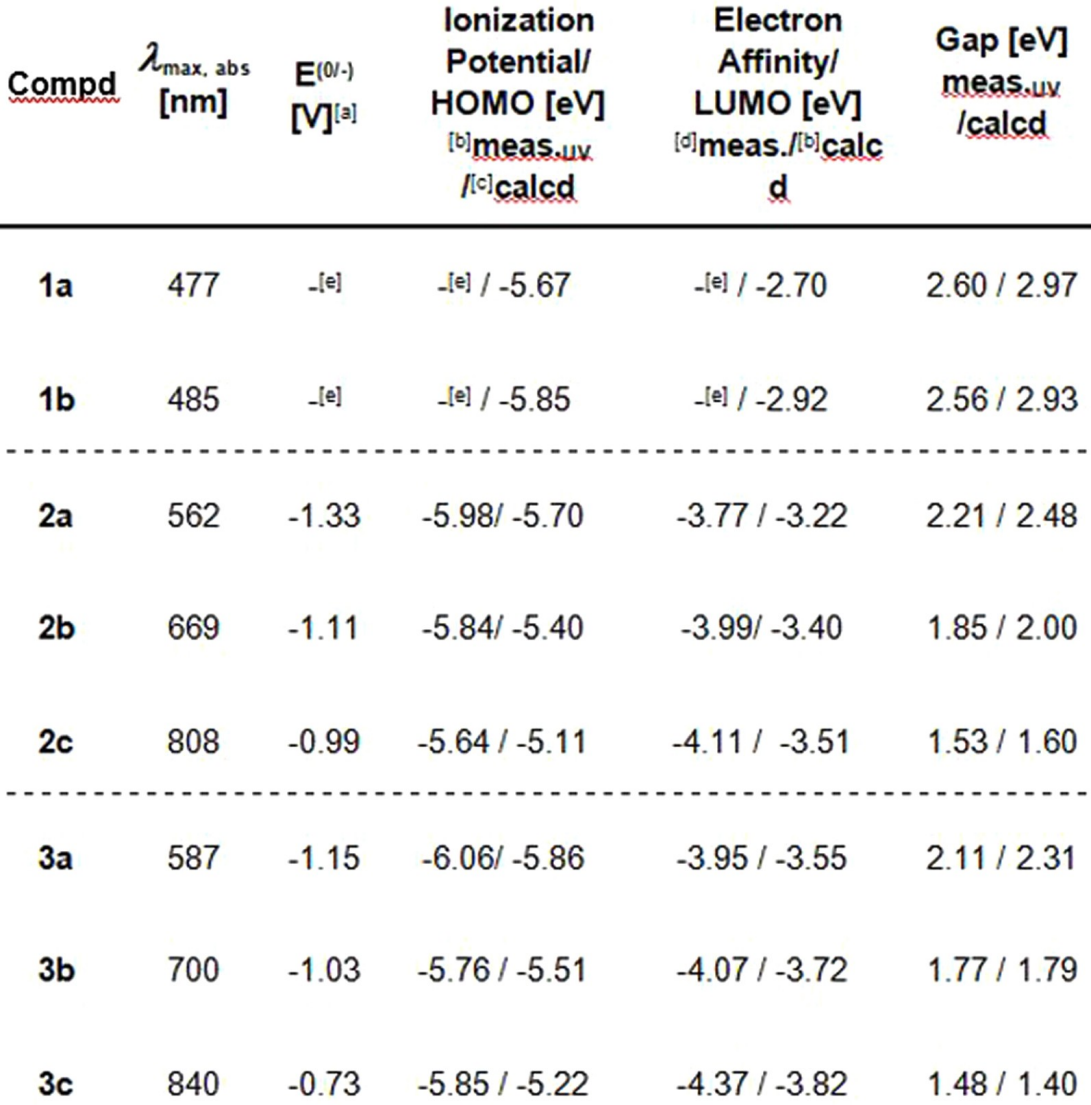
Experimental and calculated (gas‐phase) properties of annulated heteroarenes.

[a] First reduction potentials determined via cyclic voltammetry (CV) in DCM at room temperature with Bu_4_NPF_6_ as the electrolyte against Fc/Fc^+^ as an internal standard (−5.10 eV)^[33]^ at 0.1 V s^−1^, 0.2 V s^−1^ or 0.5 V s^−1^; [b] ionization potential=electron affinity−λ_onset,abs_; [c] obtained from DFT calculations (Gaussian16^[34]^ B3LYP/def2‐TZVP) [d] electron affinity=−e(5.1 V+E^(0/−)^); [e] measurement not possible due to insufficient solubility.

We grew crystals for x‐ray diffraction analyses by diffusion of methanol into a saturated solution of the arenes in chloroform or dichlormethane. The crystal structure of **2 b** and **3 b** (Figure 4) unveil that **2 b** forms a 1D staircase with overlapping acene units. The distance between the layers is estimated to 3.64 Å. Besides the π–π overlap there are also TIPS‐π‐interactions to the next 1D stack. In the crystalline state, **2 b** is 1.9 nm long (measured without hydrogen atoms).

**Figure 4:**
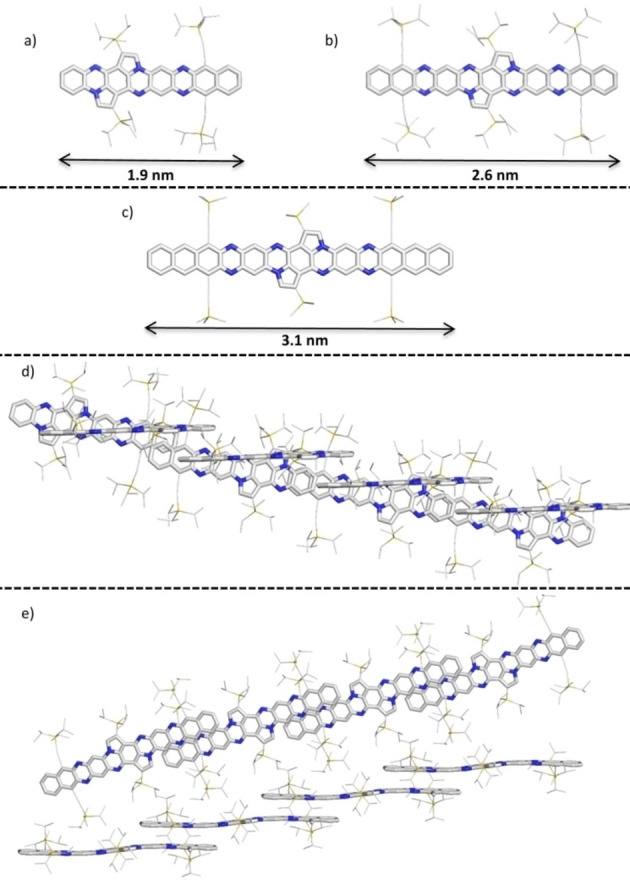
Single crystal structure of **2 b** (a) and **3 b** (b); c) calculated structure of **3 c** (Gaussian16^[34]^ B3LYP/def2‐TZVP, TMS groups were used to reduce computational cost); solid‐state packings of **2 b** (d) and **3 b** (e). Hydrogen atoms were omitted and TIPS(‐ethynyl) groups reduced in size for clarity.

Compound **3 b** shows a similar packing motif as **2 b** with additional (two sided) π–π interactions. The π–π distance amounts to 3.29 Å. The length of the molecules is determined to 2.6 nm for **3 b** (measured without hydrogen atoms using the crystal structure) and 3.1 nm for **3 c** (measured without hydrogen atoms using the optimized DFT structure).^[35]^

Due to their electrochemical properties and the possibility to obtain solvent‐free crystal structures, **2 b** and **3 b** were promising candidates for thin film transistors (TFTs). TFTs were fabricated in bottom gate/top contact architectures (Figure 5 and Supporting Information) with silver as contact electrodes, using 12‐cyclohexyldodecylphosphonic acid as self‐assembling monolayer (SAM) to modify the dielectric (SiO_2_/AlO_*x*_).^[36]^ Thin films of **2 b** and **3 b** were obtained by drop‐casting (**2 b**: toluene, 0.5 mg mL^−1^; **3 b**: DCM, 0.5 mg mL^−1^). The average electron mobility of **3 b** (*μ*_ave_=1.4×10^−3^ cm^2^ V^−1^ s^−1^) is one order of magnitude higher than that of the mono‐postfunctionalized compound **2 b** (*μ*_ave_=2.1×10^−4^ cm^2^ V^−1^ s^−1^) probably due to π–π‐interactions at the western and eastern benzene rings of **3 b** compared to **2 b**. As **3 c** did not furnish satisfactory thin‐films due to its decreased solubility compared to **3 b**, OFETs could not be obtained.

**Figure 5:**
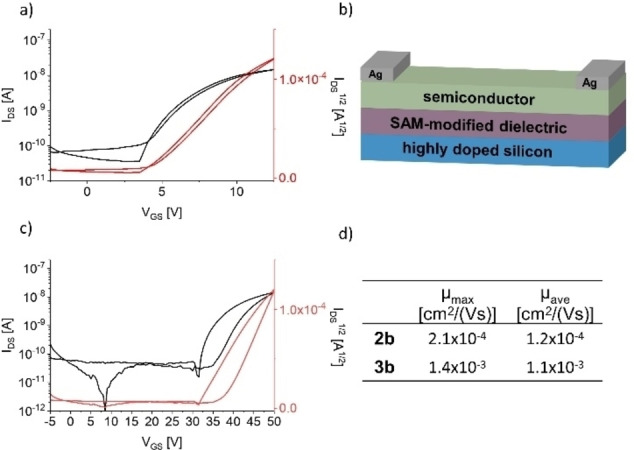
a) Transfer characteristics of **3 b**; b) schematics of the bottom‐gate/top‐contact device architecture; c) transfer characteristics of **2 b**; d) electron transfer mobilities of **2 b** and **3 b** (calculated from the forward sweep).

To verify the measured electron mobilities, we calculated transfer integrals of all different types of neighboring molecular pairs out of the crystal structure using the ADF software package.^[37]^ Reorganization energies were calculated by the four‐point method^[38]^ using Gaussian16. These values were employed for the calculation of a theoretical electron transport mobility *μ* (Table 2).^[39]^ Only the highest transfer integral of each compound is shown—all other transfer integrals, including images of the corresponding dimer pairs, are listed in the Supporting Information, Figure S19, S20. The calculated electron mobilities (**2 b**: *μ*_theo_=0.69 cm^2^ V^−1^ s^−1^; **3 b**: *μ*_theo_=2.6 cm^2^ V^−1^ s^−1^) also predict that the value for **3 b** is one order of magnitude higher compared to **2 b**. The absolute calculated mobilities differ from the measured ones, possibly due to differences in the solid state structure in the film compared to that of the single crystal.

**Table 2:**
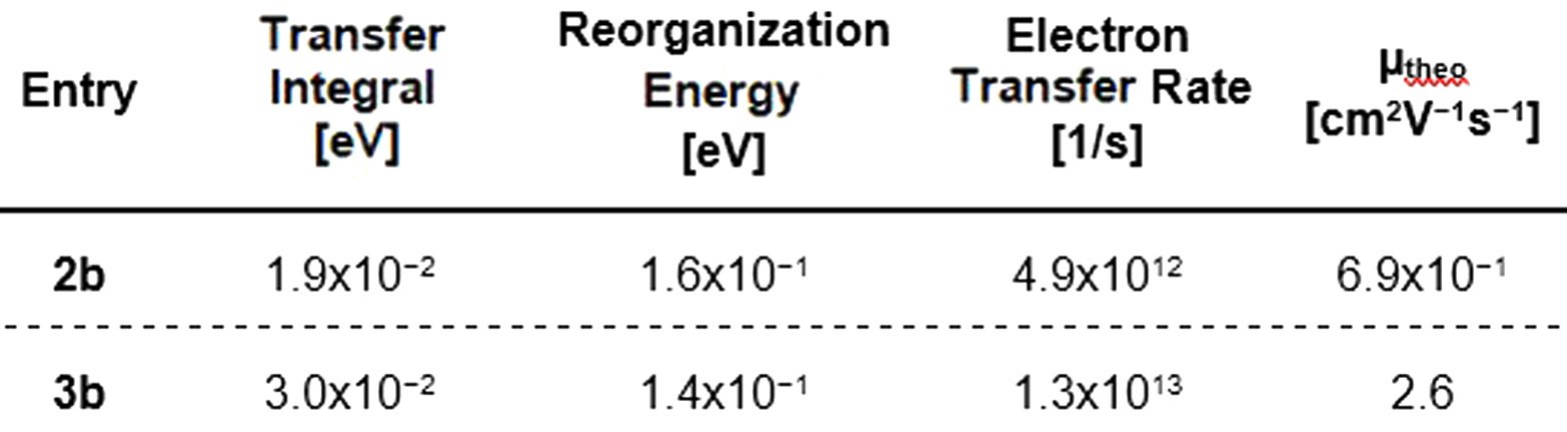
Calculated transfer integrals, reorganization energies and electron mobilities for an electron transport.

To assess the stability of the compounds in solution, time dependent absorption spectroscopy was performed on **2 b**,**c** and **3 b**,**c** in dichloromethane. We chose **TIPS‐TAP** ^[40]^ as reference (Figure 6). The solutions were irradiated with UV‐ and white light under ambient conditions (for more details, see the method section in the Supporting Information). In Figure 6 time dependent decay of the absorption intensity is depicted. All synthesized molecules are more stable than **TIPS‐TAP** (*t*_1/2_=15 min). The mono‐functionalized derivatives (**2 b**: *t*_1/2_=25 min, **2 c**: *t*_1/2_=17 min) are less stable in comparison with their difunctionalized congeners (**3 b**: *t*_1/2_=45 min, **3 c**: *t*_1/2_=35 min). The most stable material is **3 b**, whose half‐life is three times higher compared to that of **TIPS‐TAP**.

**Figure 6:**
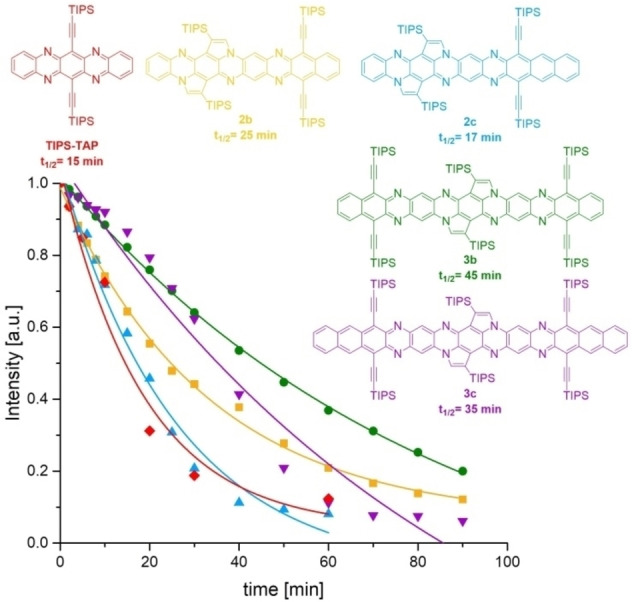
Time dependent decrease of the absorption intensity of **TIPS‐TAP** (679 nm, red), **2 b** (594 nm, yellow), **2 c** (805 nm, blue), **3 b** (700 nm, green) and **3 c** (824 nm, violet) in DCM solution under ambient conditions and irradiation with UV‐light and white light. For clarity reasons, intensity values were fitted with an exponential fit.

To characterize the electron transporting species in the n‐type OFETs, we attempted to synthesize the corresponding radical anion and dianion of **3 b** by adding 1 or 2 equivalents of potassium anthracenide to its THF solution. After addition of 1 equivalent, the deep blueish solution turned purple under N_2_. EPR measurements (Supporting Information, Figure S14) support the presence of a radical anion. Unfortunately, the compound decomposes quickly under ambient conditions to the starting material ‐ we were neither able to obtain suitable single crystals nor to measure a suitable absorption spectra. Adding another equivalent of the reducing reagent results in a color shift to orange. Again, the instability of this species under air prevented further characterization.

In conclusion we synthesized a new class of azaarenes via modular π‐extension of a cyclopentannulated azaarene core via Buchwald–Hartwig couplings. All aza‐nanocarbons are stable under ambient conditions ‐ the largest compound comprises 13 annulated rings in a row. **2 b** and **3 b** were used as semiconductors in organic field effect transistors with a bottom gate/top contact architecture. Di‐postfunctionalized **3 b** shows an electron mobility which is one order of magnitude higher compared to the mono‐postfunctionalized compound **2 b**. This is most likely the consequence of π–π‐interactions in two directions.

Deposition Numbers 2208801 (for **2b**), 2208802 (for **2c**), 2208803 (for **3b**) contain the supplementary crystallographic data for this paper. These data are provided free of charge by the joint Cambridge Crystallographic Data Centre and Fachinformationszentrum Karlsruhe Access Structures service.

## Conflict of interest

The authors declare no conflict of interest.

## Supporting information

As a service to our authors and readers, this journal provides supporting information supplied by the authors. Such materials are peer reviewed and may be re‐organized for online delivery, but are not copy‐edited or typeset. Technical support issues arising from supporting information (other than missing files) should be addressed to the authors.

Supporting Information

Supporting Information

## Data Availability

Data related to this article are available via heiDATA, the institutional research data repository of Heidelberg University, under the following DOI: 10.11588/data/CSP7AW.
