# Supplementary material for: Azaarenes: 13 Rings in a Row by Cyclopentannulation
Source: Angew Chem Int Ed Engl. 2022 Dec 22;62(5):e202214031. doi: 10.1002/anie.202214031 (PMC10107455; doi:10.1002/anie.202214031)
Supplement: Supplementary file 1 — Supporting Information [file ANIE-62-0-s001.pdf]

Angewandte Chemie
Eine Zeitschrift der Gesellschaft Deutscher Chemiker

Supporting Information

Azaarenes: 13 Rings in a Row by Cyclopentannulation

S. Maier, N. Hippchen, F. Jester, M. Dodds, M. Weber, L. Skarjan, F. Rominger, J. Freudenberg, U. H. F. Bunz**

Table of Content

1 Experimental Procedures 2
1.1 General Information 2
1.2 General Procedures 3
1.3 Synthesis of the Compounds 3
2 NMR Spectra 8
3 UV-Vis Spectra 14
4 EPR Spectrum 14
5 Crystallographic Data 15
6 Computational Details 19
6.1 Calculations of the Optoelectronic Properties 19
6.1.1 FMO Calculations 19
6.1.2 Calculation of theoretical electron mobilities 22
6.1.3 TD-DFT calculations 23
6.1.4 Coordinates of the Optimized Geometries 24
7 Cyclic Voltammetry and Dynamic Pulse Voltammetry 40
8 Device Manufacturing and Characterization 42
9 Stability Measurements 43
10 References 44

1 Experimental Procedures

1.1 General Information

Chemicals were bought from commercial suppliers (ABCR, Acros, Alfa Aesar, Carbolution, Chempur, Fluka, Merck, Sigma Aldrich and TCI) and used as delivered. Anhydrous solvents were dispensed from a solvent purification system MB SPS-800. Deuterated solvents were bought from Euriso Top and Sigma Aldrich.
Melting points (mp) were measured in open glass capillaries on a Stuart SMP10 melting point apparatus and are uncorrected.
R_f-values were determined by analytical thin-layer chromatography (TLC) on aluminum sheets coated with silica gel produced by Macherey-Nagel (ALUGRAM® Xtra SIL G/25 UV_{254}). Detection was accomplished using UV light (254 and 365 nm) or a TLC staining solution (vanillin).
Nuclear magnetic resonance (NMR) spectra were recorded at the chemistry department of Heidelberg University under the supervision of Dr. J. Graf on the following spectrometers: Bruker Avance III 300 (300 MHz), Bruker Avance DRX 300 (300 MHz), Bruker Fourier 300 (300 MHz), Bruker Avance III 400 (400 MHz), Bruker Avance III 500 (500 MHz) and Bruker Avance III 600 (600 MHz). Chemical shifts (δ) are given in ppm and coupling constants *J* in Hz. Spectra were referenced to residual solvent protons according to Fulmer *et al.*[1] The following abbreviations were used to describe the observed multiplicities: for ^{1}H NMR spectra: s = singlet, d = doublet, sept = septet, m = multiplet br = broad signal.
High-resolution mass spectra (HR-MS) were recorded at the chemistry department of Heidelberg University under the supervision of Dr. J. Gross on the following spectrometers: JEOL AccuTOF GCx (EI), Bruker ApexQe hybrid 9.4 T FT-ICR (ESI, MALDI, DART), Finnigan LCQ (ESI) and Bruker AutoFlex Speed (MALDI). For MALDI, *trans*-2-[3-(4-*tert*-butylphenyl)-2-methyl-2-propenylidene]-malononitrile (DCTB) was used as matrix.
Infrared spectra were recorded as neat powders on a FTIR spectrometer (Bruker LUMOS or Jasco FT/IR-4100) with a Germanium ATR-crystal. For the most significant bands the wavenumbers are given.
UV-Vis spectra were recorded on a Jasco UV-VIS V-670. Fluorescence spectra were recorded on a Jasco FP6500.
Preparative gel permeation chromatography (GPC) was performed on Bio-Beads® (S-X1 Beads, 200 – 400 Mesh, crosslinked polystyrene) purchased from Bio-Rad Laboratories, Inc., using toluene as eluent.
Cyclovoltammetry (CV) measurements were performed on a Metrohm Autolab PGSTAT101 with a scan rate of 0.1 V/s, 0.2 V/s or 0.5 V/s with ferrocene as internal standard. Differential pulse voltammetry (DPV) was performed on a VersaSTAT 3 potentiostat by Princeton Applied Research using ferrocene as internal standard. The DPV was obtained with a step size of 0.005 V, a modulation amplitude of 0.025 V, a modulation time of 0.05 s and an interval time of 0.5 s.
X-ray crystallography was carried out at the chemistry department of Heidelberg University under the supervision of Dr. F. Rominger on the following instruments: Bruker Smart APEX II Quazar (with Mo-microsource) and Stoe Stadivari (with Co-microsource and Pilatus detector).
For flash column chromatography silica gel (Sigma Aldrich, pore size 60 Å, 70–230 mesh, 63–200 µm) was used as stationary phase. As eluents different mixtures of petroleum ether (PE), ethyl acetate (EA), dichloromethane (DCM) or MeOH were used.
All reactions were performed under air, if not otherwise specified. For handling of air and moisture sensitive reagents, standard Schlenk techniques with flame-dried glassware under an argon or nitrogen atmosphere were used. Compounds **S1**-**S3**[2], **1a** and **1b**[3] were synthesized according to a literature procedure.

1.2 General Procedures

Experimental Section

GP1:Buchwald-Hartwig Amination towards Mono Postfunctionalized Cyclopentannulated Tetraazapentacenes

The bromoarene (1.10 eq.) and the corresponding amine (1.00 eq.) were dissolved in dry toluene under an argon atmosphere. The resulting solution was degassed for 30 min. Caesium carbonate (5.00 eq.) and RuPHOS Pd G1 (10.0 mol%.) were added and the reaction mixture was stirred at 140 °C for 20 h. After cooling to room temperature, a saturated ammonium chloride solution was added and the mixture was extracted with DCM. The combined organic layers were dried over $MgSO_4$ and the crude product was purified by flash column chromatography to yield a mixture of the product and the corresponding NH-species. This mixture was dissolved in DCM and MnO_2 (100 eq.) was added. The suspension was stirred for 1 h at room temperature. Afterwards, the mixture was filtered through Celite and the solvent was removed under reduced pressure. The almost pure compound was finally purified by GPC (toluene) to yield the pure product.

GP2: Buchwald-Hartwig Amination towards Doubly Post-functionalized Cyclopentannulated Tetraazapentacenes

The bromoarene (1.00 eq.) and the corresponding amine (2.20 eq.) were dissolved in dry toluene under an argon atmosphere. The resulting solution was degassed for 30 min. Caesium carbonate (10.0 eq.) and RuPHOS Pd G1 (20.0 mol%.) were added and the reaction mixture was stirred at 140 °C for 48 h. After cooling to room temperature, a saturated ammonium chloride solution was added and the mixture was extracted with DCM. The combined organic layers were dried over $MgSO_4$ and the crude product was purified by flash column chromatography to yield a mixture of the product and the corresponding NH-species. This mixture was dissolved in DCM and MnO_2 (100 eq.) was added. The suspension was stirred for 1 h at room temperature. Afterwards, the mixture was filtered through Celite and the solvent was removed under reduced pressure. The almost pure compound was finally purified by GPC (toluene) to yield the pure product.

1.3 Synthesis of the Compounds

Synthesis of 2,9-bis[tri(propan-2-yl)silyl]-13,16-bis([tri(propan-2-yl)silyl]ethynyl)-3,7b,10,12,17,18b-hexaazadicyclopenta[fg,yz]heptacene (2a)

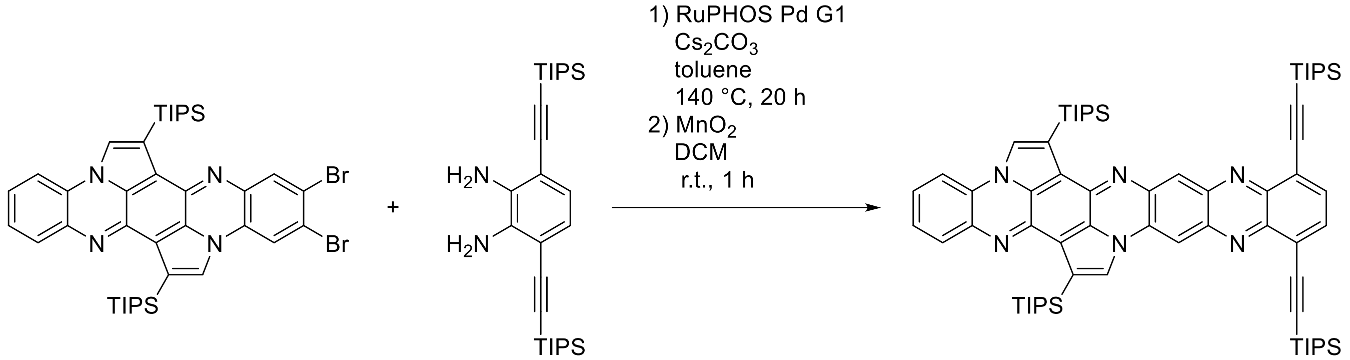

1a **S1** **2a**

GP1 was applied to **1a** (160 mg, 199 µmol, 1.10 eq.), **S1** (85.0 mg, 181 µmol, 1.00 eq), RuPHOS Pd G1 (14.8 mg, 18.1 µmol, 10.0 mol%) and caesium carbonate (295 mg, 906 µmol, 5.00 eq) in 18 mL toluene. Column chromatography (PE:DCM 10:1, 9:1) yielded the described mixture. The crude product was dissolved in 18 mL DCM and manganese(IV) oxide (1.57 g, 18.1 mmol, 100 eq.) was added. The pure compound was yielded as a red solid (74.0 mg, 66.7 µmol, 37%).
Mp: >300 °C.

***R*f**: 0.4 (silica gel, PE:DCM = 9:1).

^{1}H NMR (600 MHz, $CDCl_3$, 298 K) δ = 8.66 (s, 1H), 8.49 (s, 1H), 8.10 (s, 1H), 8.07-8.05 (m, 1H), 8.00-7.99 (m, 1H), 7.97 (s, 1H), 7.95-7.92 (m, 2H), 7.61-7.54 (m, 2H), 2.11-1.99 (m, 6H), 1.33-1.32 (m, 42H), 1.29-1.27 (m, 36H) ppm.

^{13}C NMR (151 MHz, $CDCl_3$, 298 K) δ = 148.7, 146.1, 143.9, 143.6, 143.0, 142.5, 142.5, 139.2, 133.6, 133.0, 131.2, 130.4, 129.2, 129.1, 128.0, 127.5, 126.7, 126.4, 125.0, 124.4, 124.2, 123.8, 123.1, 120.5, 119.2, 117.9, 114.4, 110.8, 104.0, 103.8, 101.3, 100.9, 19.4, 19.4, 19.0, 19.0, 12.5, 11.8, 11.7 ppm.

ATR-IR: $\tilde{\nu}$ [cm^{-1}] = 2940, 2923, 2888, 2862, 2362, 2359, 2342, 2337, 2330, 1502, 1490, 1458, 1426, 1382, 1367, 1292, 1249, 1226, 1191, 1122, 1105, 1039, 1019, 996, 881, 848, 798, 791, 776, 763, 748, 735, 677, 669, 663, 643, 620, 590, 581, 576, 551, 546, 505, 491, 481, 476, 462, 457, 446, 419, 411, 407.

HR-MS (MALDI pos.) *m/z*: [M+H]$^+$: calcd. for $[C_{68}H_{85}N_6Si_4]^+$: 1107.6690; found: 1107.6702.

Synthesis of 2,9-bis[tri(propan-2-yl)silyl]-13,18-bis([tri(propan-2-yl)silyl]ethynyl)-3,7b,10,12,19,20b-hexaazadicyclopenta[fg,c1d1]octacene (2b)

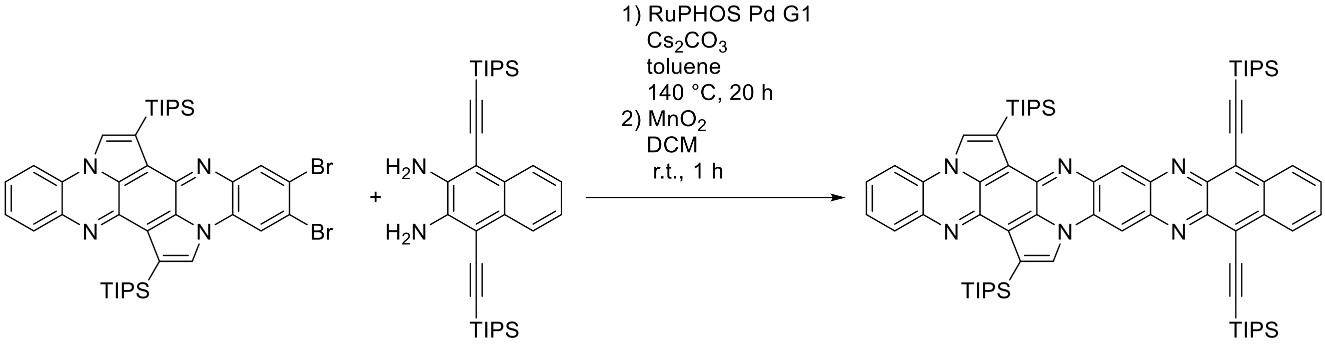

1a **S2** **2b**

GP 1 was applied to **1a** (119 mg, 148 µmol, 1.10 eq.), **S2** (70.0 mg, 135 µmol, 1.00 eq), RuPHOS Pd G1 (11.0 mg, 13.5 µmol, 10.0 mol%) and caesium carbonate (220 mg, 674 µmol, 5.00 eq) in 15 mL toluene. Column chromatography (PE:DCM 10:1, 9:1) yielded the described mixture. The crude product was dissolved in 15 mL DCM and manganese(IV) oxide (1.29 g, 14.8 mmol, 100 eq.) was added. The pure compound was yielded as a blue solid (127 mg, 110 µmol, 81%).

Mp: >300 °C.

***R*f**: 0.4 (silica gel, PE:DCM = 9:1).

^{1}H NMR (600 MHz, $CDCl_3$, 298 K) δ = 8.74-8.72 (m, 2H), 8.63 (s, 1H), 8.43 (s, 1H), 8.09-8.07 (m, 2H), 8.02-8.00 (m, 1H), 7.97 (s, 1H), 7.66-7.64 (m, 2H), 7.61-7.57 (m, 2H), 2.12-2.07 (m, 3H), 2.04-1.99 (m, 3H), 1.39-1.38 (m, 42H), 1.29-1.27 (m, 36H) ppm.

^{13}C NMR (151 MHz, $CDCl_3$, 298 K) δ = 148.6, 146.0, 143.8, 143.7, 143.6, 141.9, 141.6, 139.3, 135.3, 135.1, 131.7, 130.5, 129.1, 129.0, 128.2, 128.1, 127.9, 127.8, 127.7, 127.2, 126.7, 126.5, 125.3, 124.2, 123.1, 120.7, 120.6, 120.3, 119.5, 117.9, 114.4, 110.3, 108.5, 108.0, 103.3, 103.2, 19.5, 19.4, 19.2, 19.1, 12.5, 12.5, 11.9, 11.8 ppm.

ATR-IR: $\tilde{\nu}$ [cm^{-1}] = 2958, 2939, 2886, 2861, 1541, 1501, 1490, 1470, 1456, 1438, 1426, 1408, 1382, 1367, 1341, 1295, 1289, 1234, 1227, 1215, 1192, 1173, 1153, 1136, 1098, 1072, 1044, 1021, 993, 927, 920, 915, 904, 880, 853, 844, 816, 776, 766, 761, 751, 746, 738, 716, 700, 676, 667, 652, 638, 619, 612, 602, 595, 582, 562, 554, 508, 499, 488, 480, 473, 465, 460, 452, 442, 437, 430, 424, 415, 409, 403.

HR-MS (MALDI pos.) *m/z*: [M]$^+$: calcd. for [$C_{72}H_{96}N_6Si_4$]$^+$: 1156.6768; found: 1156.6752.

Synthesis of 2,9-bis[tri(propan-2-yl)silyl]-13,20-bis([tri(propan-2-yl)silyl]ethynyl)-3,7b,10,12,21,22b-hexaazadicyclopenta[fg,g1h1]nonacene (2c)

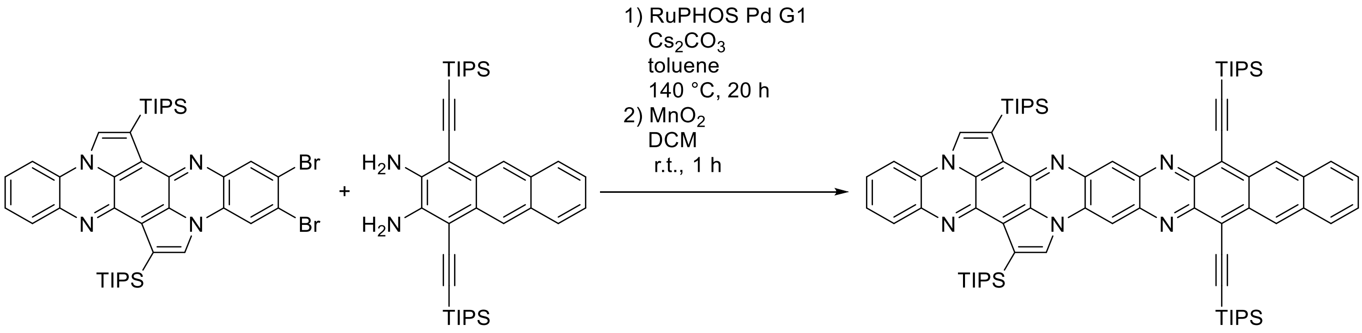

1a **S3** **2c**

GP 1 was applied to **1a** (110 mg, 137 µmol, 1.10 eq.), **S3** (70.9 mg, 125 µmol, 1.00 eq), RuPHOS Pd G1 (10.0 mg, 12.5 µmol, 10.0 mol%) and caesium carbonate (203 mg, 623 µmol, 5.00 eq) in 15 mL toluene. Column chromatography (PE:DCM 10:1, 9:1) yielded the described mixture. The crude product was dissolved in 15 mL DCM and manganese(IV) oxide (1.08 g, 12.5 mmol, 100 eq.) was added. The pure compound was yielded as a blue solid (71 mg, 58.7 µmol, 47%).

Mp: >300 °C.

***R*$_\mathbf{f}$**: 0.3 (silica gel, PE:DCM = 9:1).

^{1}H NMR (600 MHz, $CDCl_3$, 298 K) δ = 9.40 (s, 2H), 8.57 (s, 1H), 8.37 (s, 1H), 8.10-8.01 (m, 5H), 7.98 (s, 1H), 7.62-7.58 (m, 2H), 7.49-7.48 (m, 2H), 2.12-2.07 (m, 3H), 2.04-1.99 (m, 3H), 1.44-1.43 (m, 42H), 1.29-1.27 (m, 36H) ppm.

^{13}C NMR (151 MHz, $CDCl_3$, 298 K) δ = 148.6, 145.9, 144.3, 144.2, 143.9, 141.7, 141.4, 139.3, 133.1, 133.0, 132.7, 132.5, 132.0, 130.5, 129.1, 128.9, 128.9, 128.9, 128.2, 127.2, 126.9, 126.9, 126.8, 126.8, 126.7, 126.5, 125.5, 124.4, 123.2, 120.6, 120.5, 120.0, 119.6, 118.0, 114.4, 110.3, 109.9, 109.3, 104.2, 104.1, 19.5, 19.4, 19.2, 19.2, 12.5, 12.5, 12.0, 11.9 ppm.

ATR-IR: $\tilde{\nu}$ [cm^{-1}] = 2957, 2941, 2888, 2864, 2367, 2362, 2358, 2344, 2334, 2328, 2323, 2174, 1539, 1502, 1490, 1472, 1463, 1446, 1425, 1388, 1378, 1364, 1300, 1222, 1196, 1181, 1175, 1154, 1141, 1104, 1098, 1019, 880, 747, 732, 678, 669, 647, 585, 512, 461, 453, 426.

HR-MS (MALDI pos.) *m/z*: [M+H]$^+$: calcd. for [$C_{76}H_{99}N_6Si_4$]$^+$: 1207.7003; found: 1207.7005.

Synthesis of 1,12-bis[tri(propan-2-yl)silyl]-5,8,16,19-tetrakis([tri(propan-2-yl)silyl]ethynyl)-2a,4,9,11,13a,15,20,22-octaazadicyclopenta[jk,c1d1]nonacene (3a)

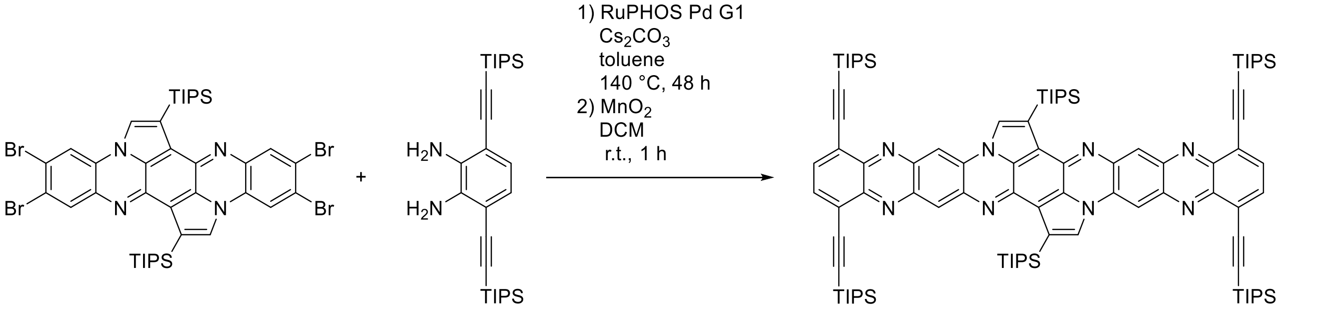

1b **S1** **3a**

GP2 was applied to **1b** (100 mg, 104 µmol, 1.00 eq.), **S1** (107 mg, 229 µmol, 2.20 eq), RuPHOS Pd G1 (17.0 mg, 20.8 µmol, 20.0 mol%) and caesium carbonate (339 mg, 1.04 µmol, 10.0 eq) in 15 mL toluene. Column chromatography (PE:DCM 10:1, 9:1) yielded the described mixture. The crude product was dissolved in 15 mL DCM and manganese(IV) oxide (905 mg, 10.4 mmol, 100 eq.) was added. The pure compound was yielded as a violet solid (35 mg, 22.3 µmol, 21%).

Mp: >300 °C.

***R*$_f$**: 0.3 (silica gel, PE:DCM = 9:1).

^{1}H NMR (700 MHz, $CDCl_3$, 298 K) δ = 8.83 (s, 2H), 8.60 (s, 2H), 8.15 (s, 2H), 7.97-7.95 (m, 4H), 2.11-2.07 (m, 6H), 1.34-1.26 (m, 120H) ppm.

^{13}C NMR (176 MHz, $CDCl_3$, 298 K) δ = 148.1, 144.0, 144.0, 142.5, 142.4, 142.0, 133.3, 130.6, 129.0, 128.6, 125.4, 124.6, 124.3, 123.3, 119.2, 111.4, 103.9, 103.6, 101.7, 101.2, 19.5, 19.1, 19.0, 12.6, 11.8, 11.8 ppm.

ATR-IR: $\tilde{\nu}$ [cm^{-1}] = 2959, 2954, 2943, 2891, 2865, 2358, 2344, 2337, 2332, 2325, 1634, 1559, 1521, 1516, 1507, 1489, 1471, 1464, 1428, 1121, 1039, 1021, 883, 758, 753, 679, 669, 663, 652, 648, 567, 553, 536, 518, 514, 509, 501, 491, 487, 483, 476, 470, 461, 451, 440, 431, 424, 415, 412.

HR-MS (MALDI pos.) *m/z*: [M]$^+$: calcd. for $[C_{96}H_{136}N_8Si_6]^+$: 1568.9498; found: 1568.9492.

Synthesis of 1,14-bis[tri(propan-2-yl)silyl]-5,10,18,23-tetrakis([tri(propan-2-yl)silyl]ethynyl)-2a,4,11,13,15a,17,24,26-octaazadicyclopenta[lm,i1j1]undecacene (3b)

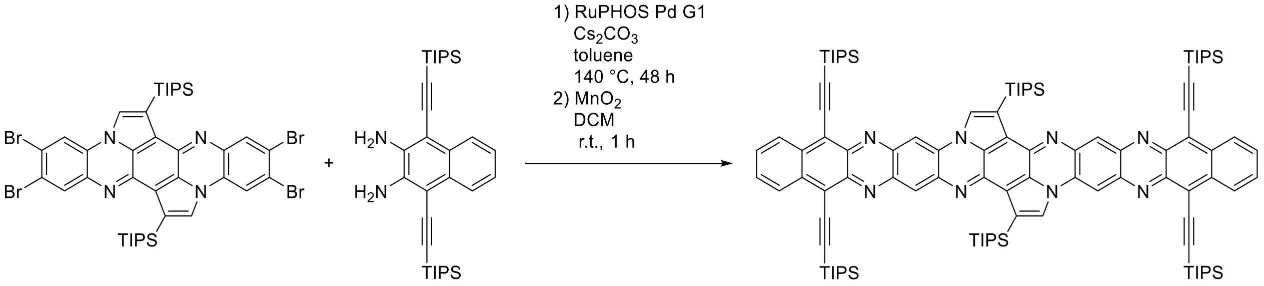

1b **S2** **3b**

GP2 was applied to **1b** (514 mg, 535 µmol, 1.00 eq.), **S2** (611 mg, 1.18 mmol, 2.20 eq), RuPHOS Pd G1 (87.4 mg, 107 µmol, 20.0 mol%) and caesium carbonate (1.74 g, 5.35 mmol, 10.0 eq.) in 50 mL toluene. Column chromatography (PE:DCM 8:2, 75:25, 7:3) yielded the described mixture. The crude product was dissolved in 50 mL DCM and manganese(IV) oxide (4.65 g, 53.5 mmol, 100 eq.) was added. The pure compound was yielded as a blue solid (471 mg, 282 µmol, 53%).

Mp: >300 °C.

***R*$_f$**: 0.55 (silica gel, PE:DCM = 7:3).

^{1}H NMR (700 MHz, $CDCl_3$, 298 K) δ = 8.83 (s, 2H), 8.75-8.74 (m, 4H), 8.57 (s, 2H), 8.16 (s, 2H), 7.86-7.66 (m, 4H), 2.11-2.07 (m, 6H), 1.41-1.33 (m, 120H) ppm.

^{13}C NMR (176 MHz, $CDCl_3$, 298 K) δ = 147.8, 143.4, 143.0, 142.8, 141.8, 141.7, 135.5, 135.1, 130.9, 128.9, 128.3, 128.2, 128.0, 127.8, 127.7, 125.6, 123.6, 120.9, 120.3, 119.1, 110.9, 108.9, 108.3, 103.0, 102.9, 19.3, 19.0, 19.0, 12.5, 11.7, 11.7 ppm.

ATR-IR: $\tilde{\nu}$ [cm^{-1}] = 2939, 2862, 1485, 1462, 1456, 1450, 1422, 1390, 1190, 1102, 1046, 920, 913, 881, 856, 763, 755, 741, 676, 673, 668, 666, 660, 656, 651, 646, 644, 640, 637, 629, 628, 617, 596, 584, 581, 580, 578, 576, 574, 573, 571, 570, 568, 567, 566, 561, 512, 509, 507, 506, 502, 498, 492, 488, 485, 482, 479, 477, 475, 473, 471, 468, 466, 464, 462, 458, 455, 453, 449, 446, 445, 443, 441, 438, 436, 434, 431, 427, 425, 422, 418, 414, 412, 409, 406.

HR-MS (MALDI pos.) *m/z*: $[M]^+$: calcd. for $[C_{104}H_{140}N_8Si_6]^+$: 1668.9811; found: 1668.9794.

Synthesis of 1,16-bis[tri(propan-2-yl)silyl]-5,12,20,27-tetrakis([tri(propan-2-yl)silyl]ethynyl)-2a,4,13,15,17a,19,28,30-octaazadicyclopenta[no,o1p1]tridecacene (3c)

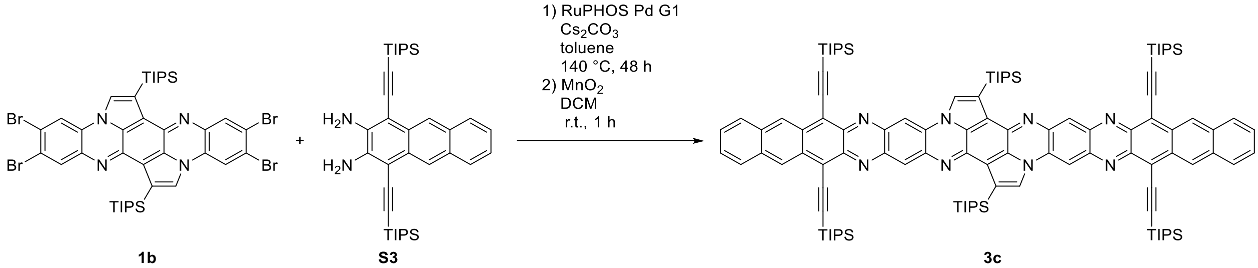

GP2 was applied to **1b** (175 mg, 182 µmol, 1.00 eq.), **S3** (228 mg, 401 µmol, 2.20 eq), RuPHOS Pd G1 (29.7 mg, 36.4 µmol, 20.0 mol%) and caesium carbonate (594 mg, 1.82 mmol, 10.0 eq.) in 30 mL toluene. Column chromatography (PE:DCM 8:2, 75:25, 7:3) yielded the described mixture. The crude product was dissolved in 50 mL DCM and manganese(IV) oxide (1.58 g, 18.2 mmol, 100 eq.) was added. The pure compound was yielded as a dark blue solid (119 mg, 67.3 µmol, 37%).

Mp: >300 °C.

***R*$_f$**: 0.55 (silica gel, PE:DCM = 7:3).

^{1}H NMR (600 MHz, $CDCl_3$, 298 K) δ = 9.42 (s, 4H), 8.77 (s, 2H), 8.50 (s, 2H), 8.14 (s, 2H), 8.07-8.04 (m, 4H), 7.51-7.49 (m, 4H), 2.12-2.07 (m, 6H), 1.45-1.44 (m, 84H), 1.35-1.34 (m, 36H) ppm.

^{13}C NMR (176 MHz, $CDCl_3$, 298 K) δ = 147.9, 144.0, 143.6, 143.2, 141.8, 141.5, 133.3, 133.1, 133.0, 132.7, 131.3, 129.2, 129.0, 128.9, 128.4, 127.1, 127.1, 127.0, 126.9, 125.9, 124.0, 120.9, 120.2, 119.3, 111.0, 110.5, 109.8, 104.2, 104.1, 19.5, 19.2, 19.2, 12.0, 11.9 ppm.

ATR-IR: $\tilde{\nu}$ [cm^{-1}] = 2942, 2925, 2890, 2865, 2366, 2358, 2344, 2337, 2331, 2325, 1628, 1559, 1521, 1507, 1486, 1462, 1424, 1381, 1242, 1196, 1175, 1137, 1096, 1073, 1060, 1031, 1019, 996, 916, 881, 753, 742, 732, 687, 676, 669, 665, 659, 653, 648, 638, 627, 615, 596, 592, 586, 581, 577, 571, 564, 518, 509, 499, 490, 485, 482, 476, 468, 461, 453, 447, 436, 431, 427, 419, 405.

HR-MS (MALDI pos.) *m/z*: $[M]^+$: calcd. for $[C_{112}H_{144}N_8Si_6]^+$: 1769.0124; found: 1769.0152.

2 NMR Spectra

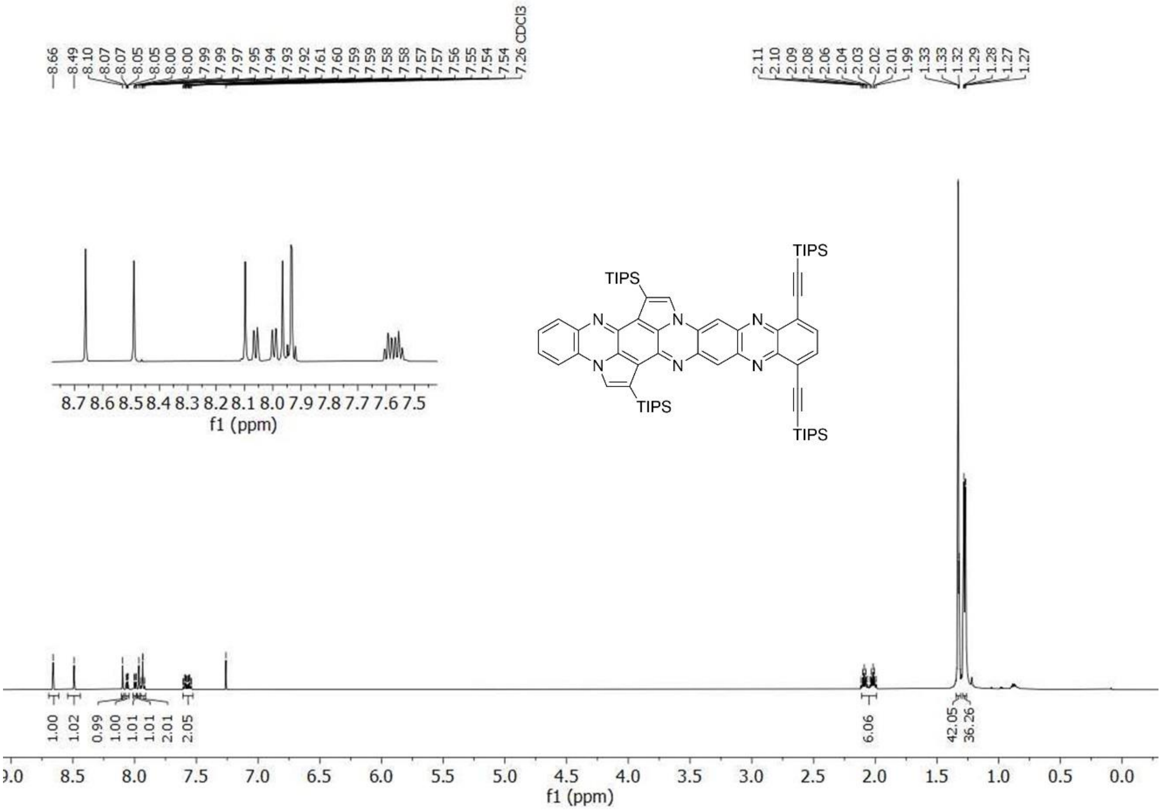

Figure S1. ^{1}H NMR spectrum (600 MHz, $CDCl_3$, 298 K) of **2a**.

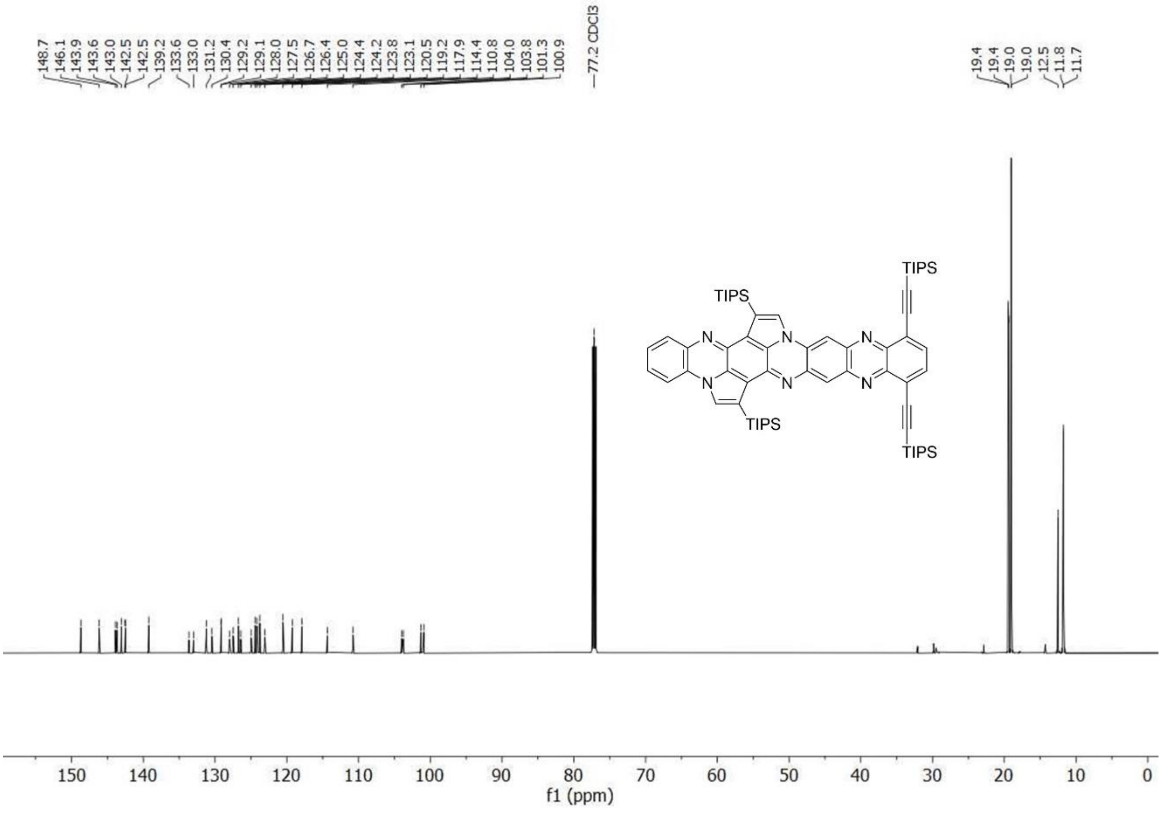

Figure S2. $^{13}C\{^{1}H\}$ NMR spectrum (151 MHz, $CDCl_3$, 298 K) of **2a**.

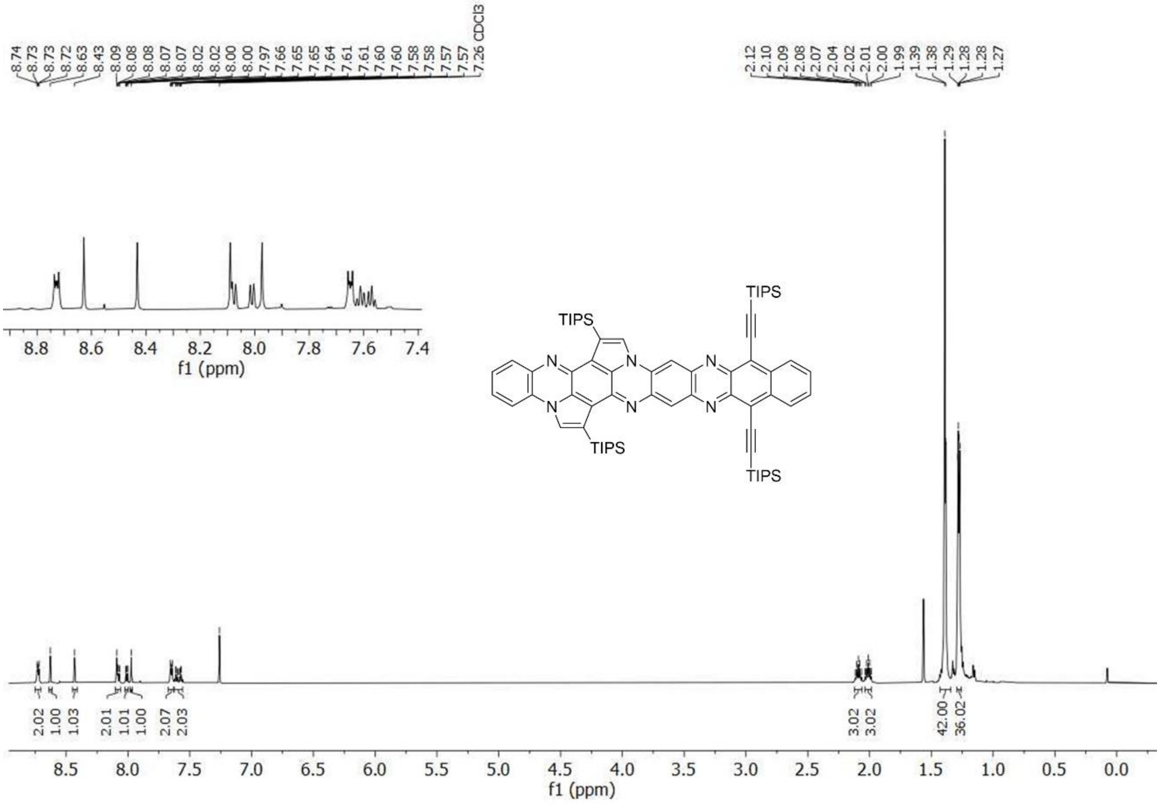

Figure S3. ^{1}H NMR spectrum (600 MHz, $CDCl_3$, 298 K) of **2b**.

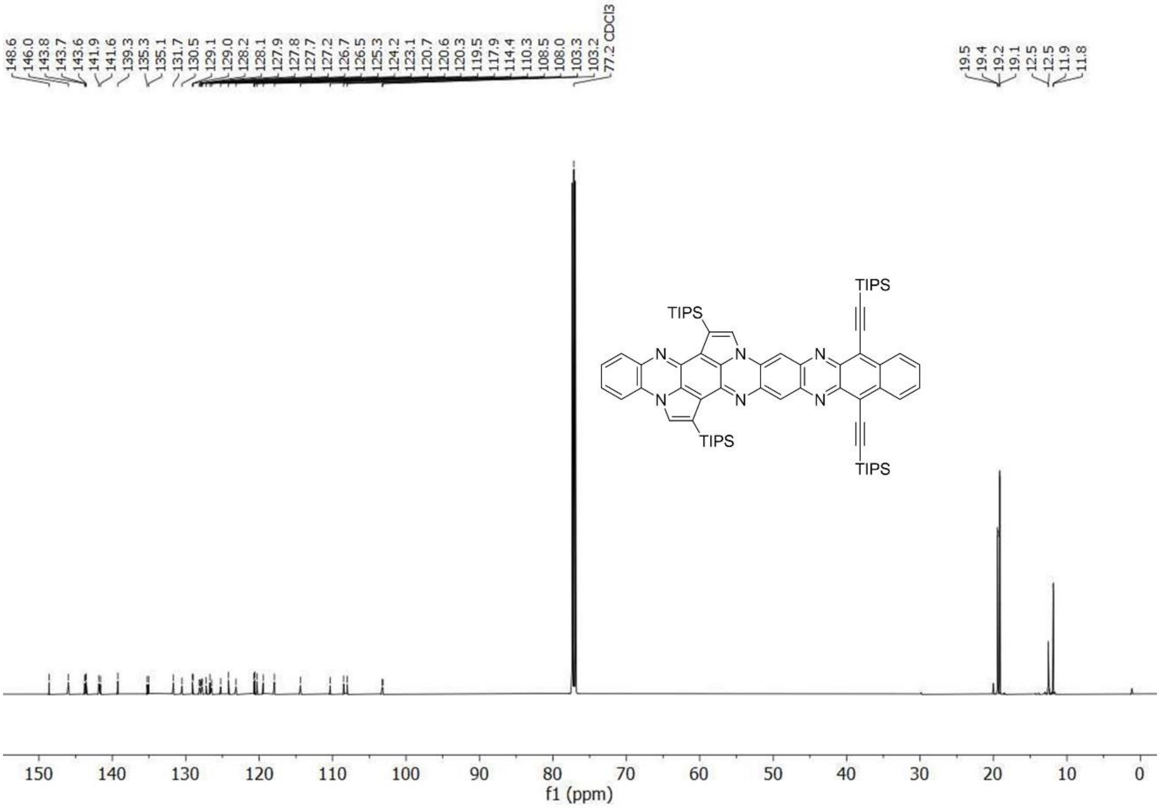

Figure S4. $^{13}C\{^{1}H\}$ NMR spectrum (151 MHz, $CDCl_3$, 298 K) of **2b**.

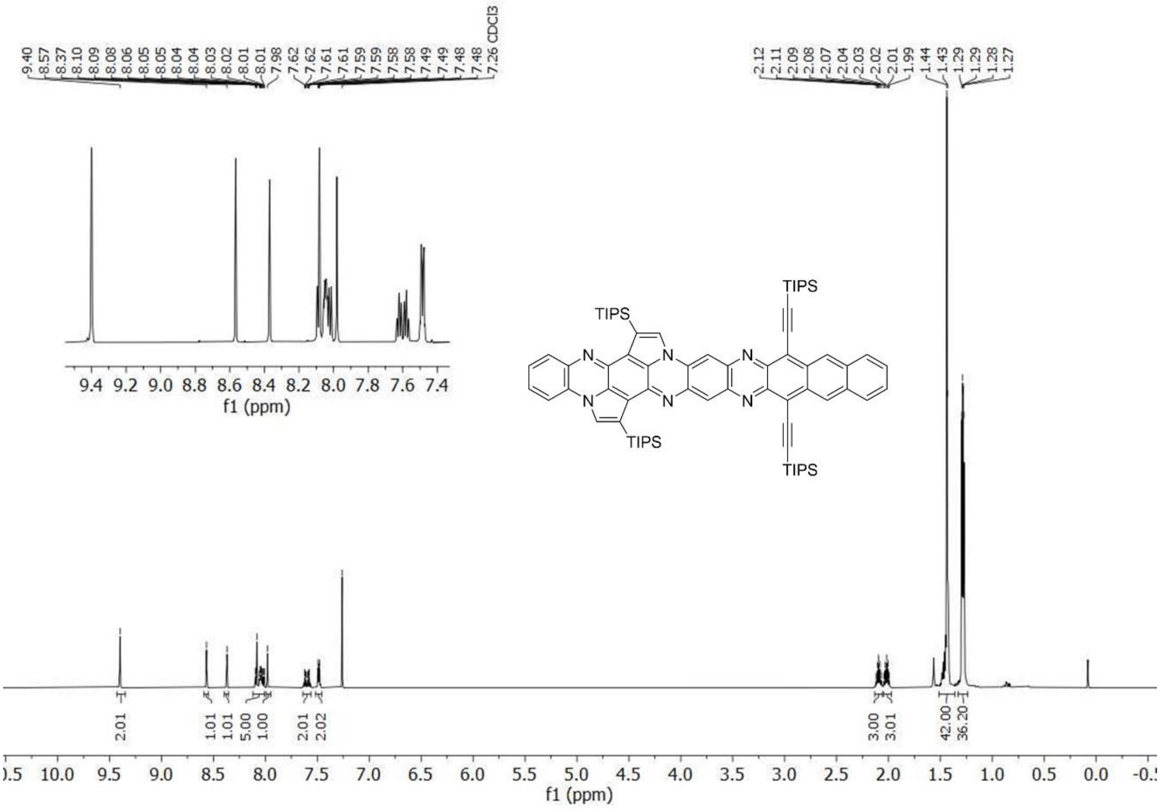

Figure S5. ^{1}H NMR spectrum (600 MHz, $CDCl_3$, 298 K) of **2c**.

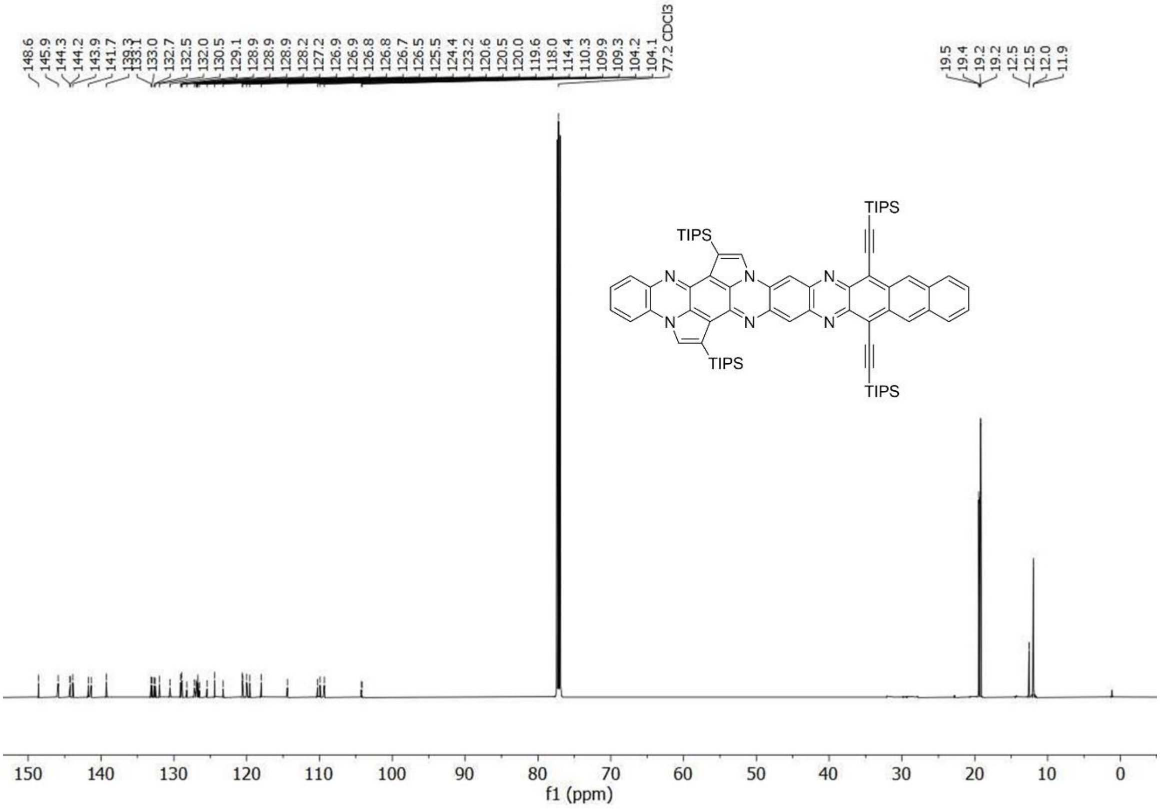

Figure S6. $^{13}C\{^{1}H\}$ NMR spectrum (151 MHz, $CDCl_3$, 298 K) of **2c**.

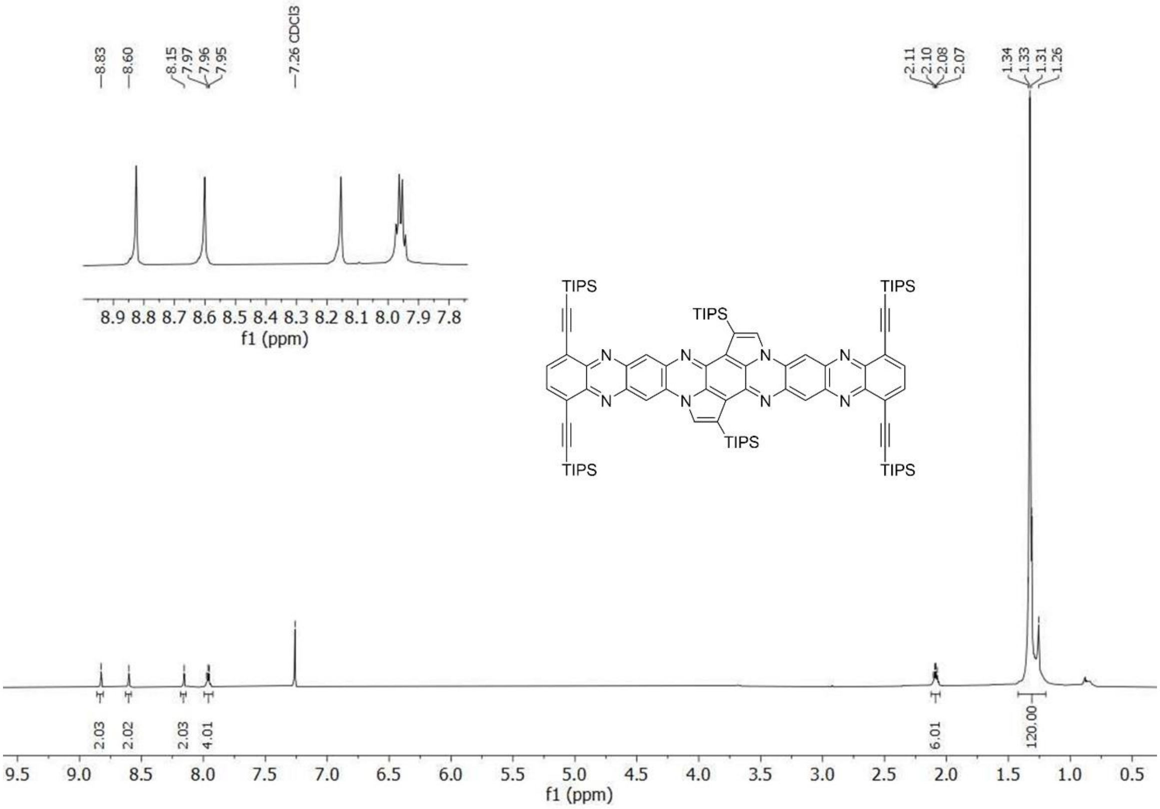

Figure S7. ^{1}H NMR spectrum (700 MHz, $CDCl_3$, 298 K) of **3a**.

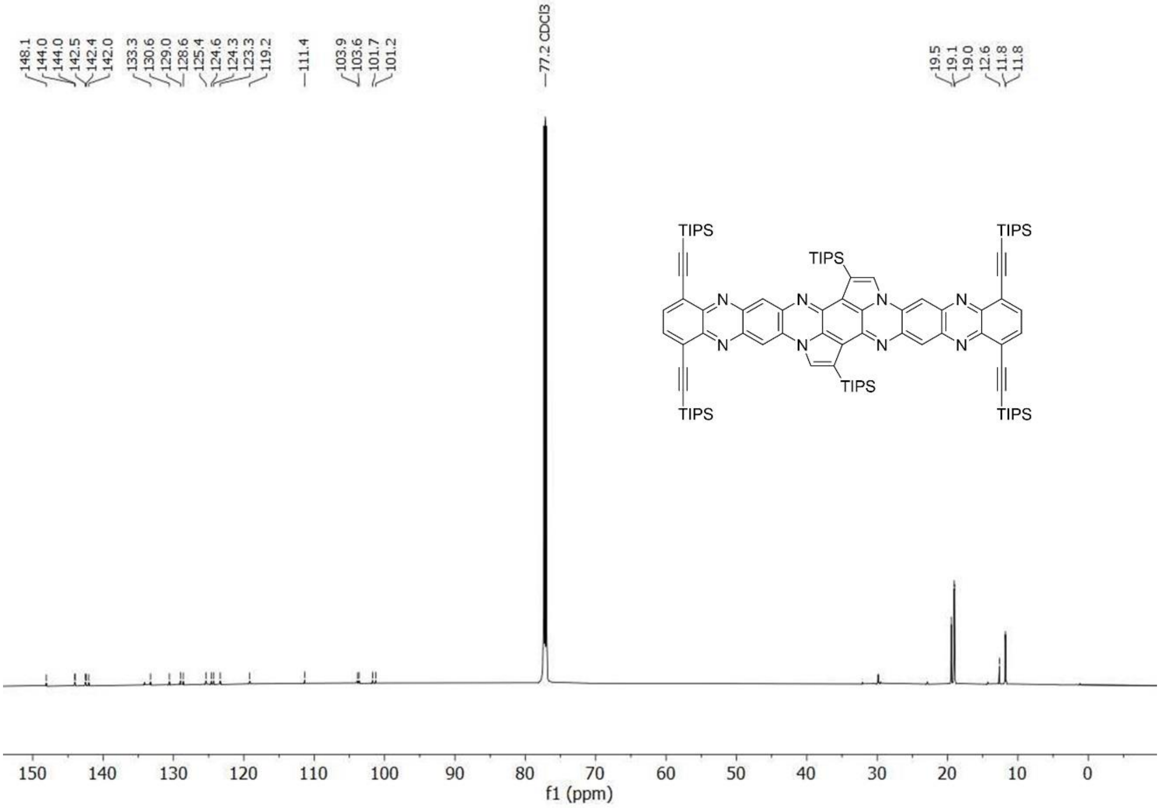

Figure S8. $^{13}C\{^{1}H\}$ NMR spectrum (176 MHz, $CDCl_3$, 298 K) of **3a**.

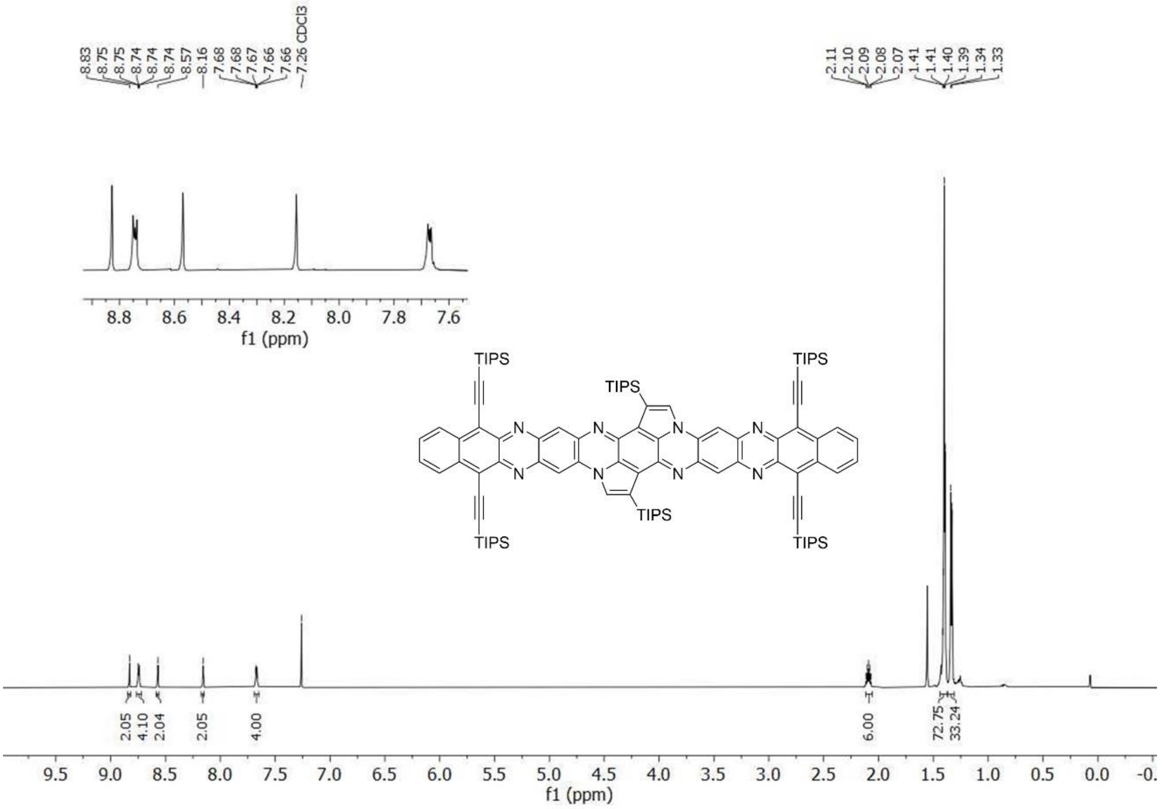

Figure S9. ^{1}H NMR spectrum (700 MHz, $CDCl_3$, 298 K) of **3b**.

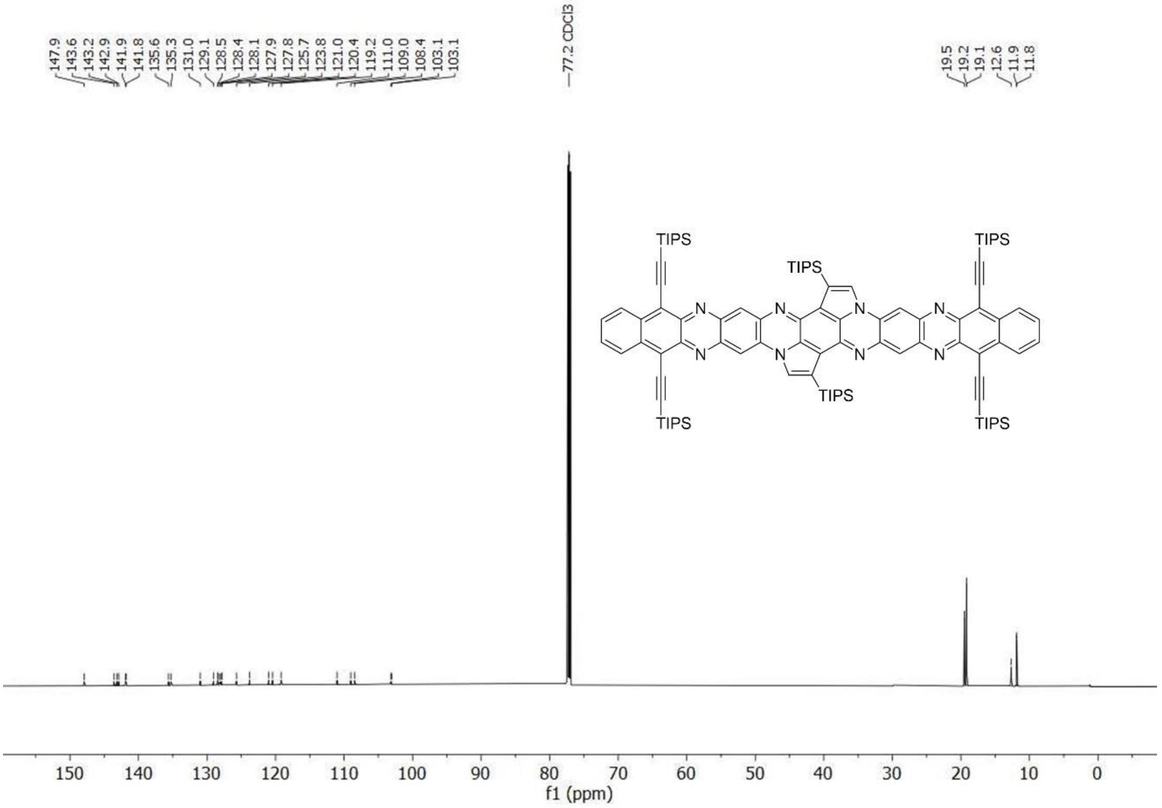

Figure S10. $^{13}C\{^{1}H\}$ NMR spectrum (176 MHz, $CDCl_3$, 298 K) of **3b**.

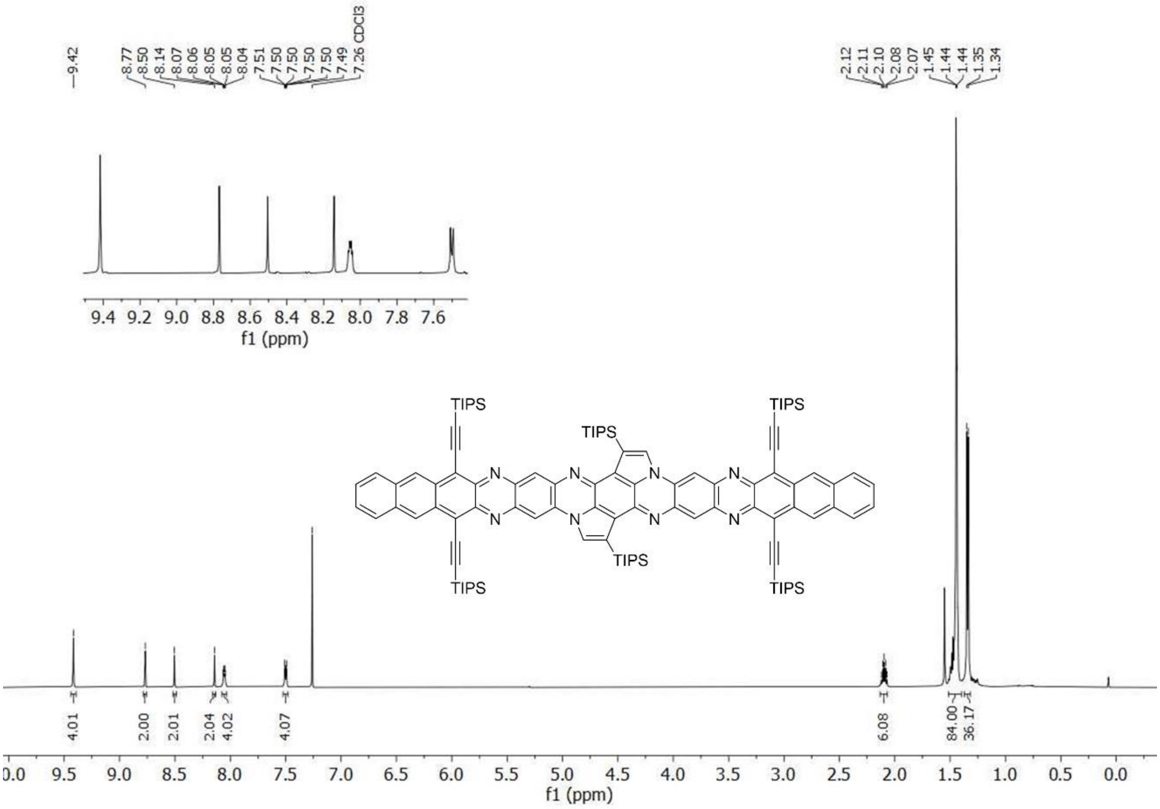

Figure S11. ^{1}H NMR spectrum (600 MHz, $CDCl_3$, 298 K) of **3c**.

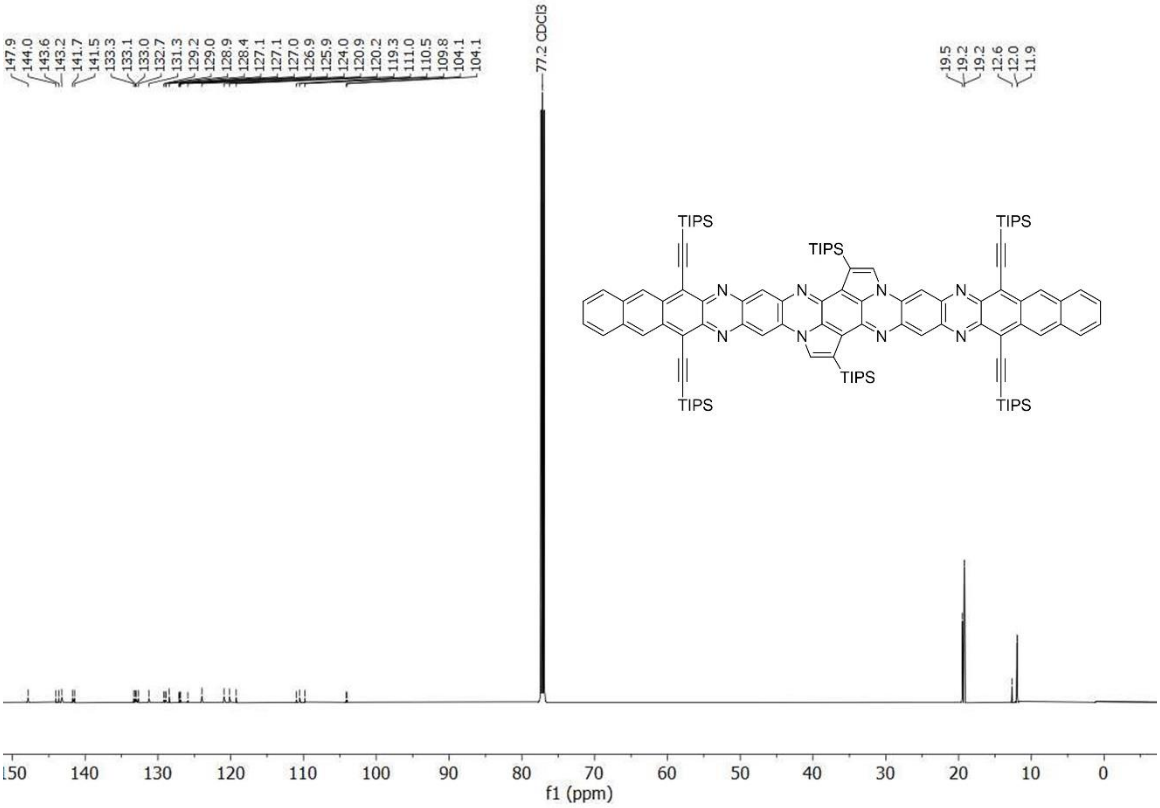

Figure S12. $^{13}C\{^{1}H\}$ NMR spectrum (151 MHz, $CDCl_3$, 298 K) of **3c**.

3 UV-Vis Spectra

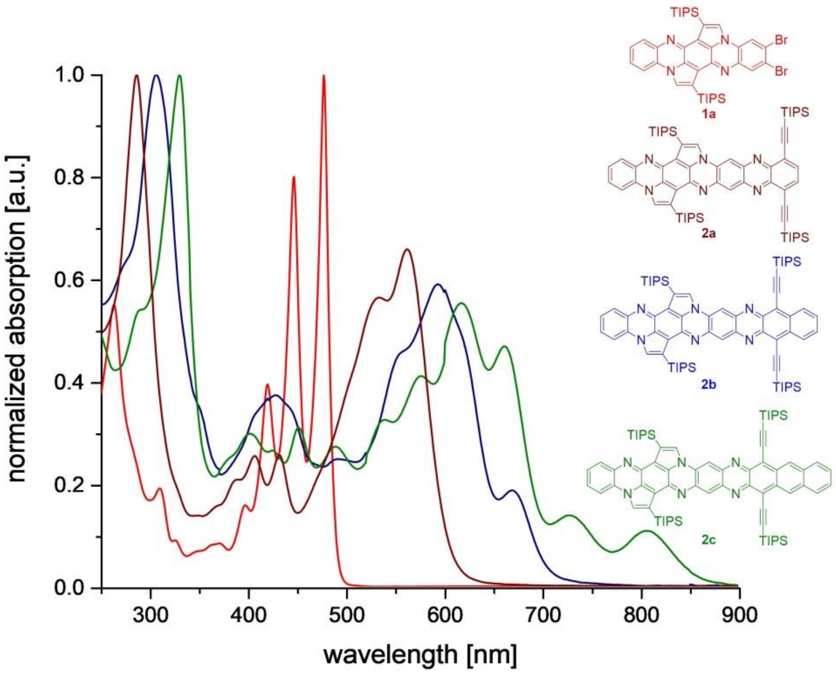

Figure S13. Normalized absorption spectra of **1a**, **2a**, **2b** and **2c** in DCM.

4 EPR Spectrum

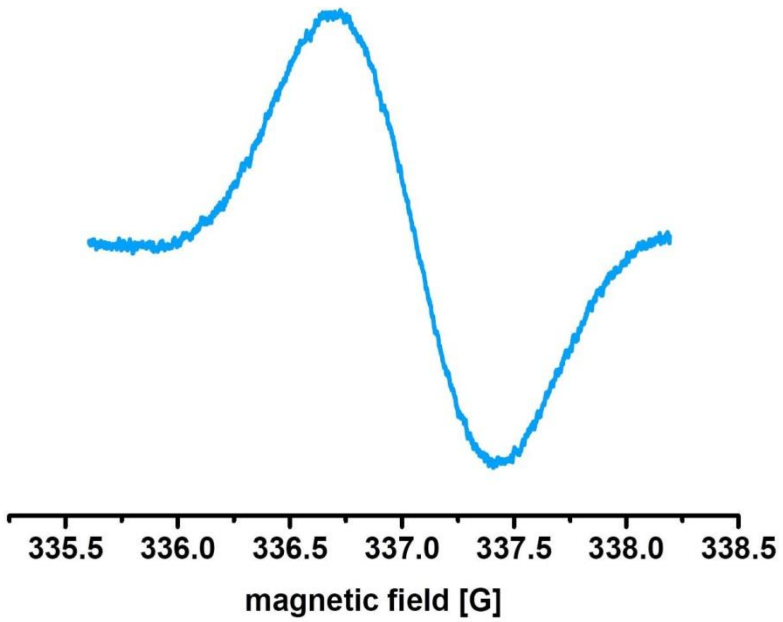

Figure S14. EPR spectrum of the radical anion of **3b** in THF.

5 Crystallographic Data

Table S1. Crystal structure, crystal data and structure refinement of **2b** (CCDC 2208801).

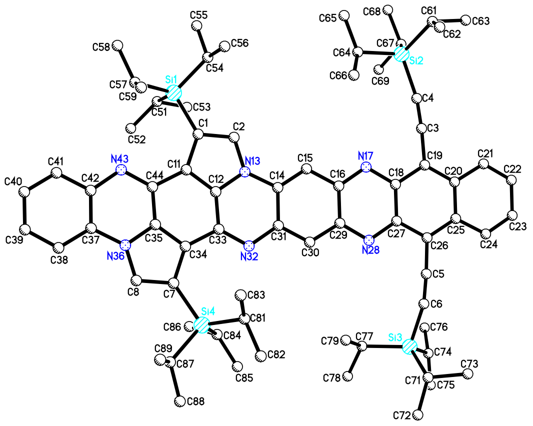

Identification code	mai47	
Empirical formula	$C_{73}H_{97}Cl_3N_6Si_4$	
Formula weight	1277.27	
Temperature	200(2) K	
Wavelength	1.54178 Å	
Crystal system	monoclinic	
Space group	$P2_1/n$	
Z	4	
Unit cell dimensions	a = 15.6934(4) Å	α = 90 deg.
	b = 26.8153(9) Å	β =113.775(2) deg.
	c = 19.0758(5) Å	γ = 90 deg.
Volume	7346.3(4) $Å^3$	
Density (calculated)	1.15 g/cm^3	
Absorption coefficient	2.08 mm^{-1}	
Crystal shape	column	
Crystal size	0.284 x 0.047 x 0.030 mm^3	
Crystal colour	violet	
Theta range for data collection	3.0 to 72.0 deg.	
Index ranges	$-19 \le h \le 9$, $-33 \le k \le 27$, $-22 \le l \le 23$	
Reflections collected	50636	
Independent reflections	13855 (R(int) = 0.0614)	
Observed reflections	8991 ($I > 2\sigma(I)$)	
Absorption correction	Semi-empirical from equivalents	
Max. and min. transmission	0.97 and 0.53	
Refinement method	Full-matrix least-squares on F^2	
Data/restraints/parameters	13855 / 5624 / 1150	
Goodness-of-fit on F^2	1.09	
Final R indices (I>2sigma(I))	R1 = 0.101, wR2 = 0.264	
Largest diff. peak and hole	0.80 and -0.44 $eÅ^{-3}$	

Table S2. Crystal structure, crystal data and structure refinement of **2c** (CCDC 2208802).

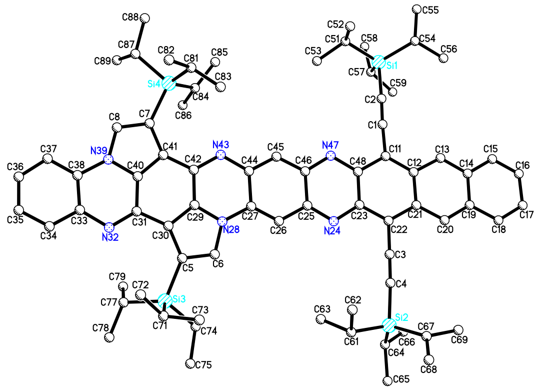

Identification code	mai59	
Empirical formula	$C_{77.50}H_{99.50}Cl_{4.50}N_6Si_4$	
Formula weight	1387.01	
Temperature	140(2) K	
Wavelength	1.54178 Å	
Crystal system	triclinic	
Space group	$P\bar{1}$	
Z	4	
Unit cell dimensions	a = 16.9399(8) Å	α =88.105(4) deg.
	b = 17.6388(8) Å	β =17.6388(8) deg.
	c = 27.0517(14) Å	γ =73.855(4) deg.
Volume	7747.6(7) $Å^3$	
Density (calculated)	1.19 g/cm^3	
Absorption coefficient	2.48 mm^{-1}	
Crystal shape	brick	
Crystal size	0.072 x 0.035 x 0.032 mm^3	
Crystal colour	violet	
Theta range for data collection	3.1 to 55.0 deg.	
Index ranges	$-17 \le h \le 17$, $-18 \le k \le 18$, $-28 \le l \le 28$	
Reflections collected	74544	
Independent reflections	18792 (R(int) = 0.0971)	
Observed reflections	5286 ($I > 2\sigma(I)$)	
Absorption correction	Semi-empirical from equivalents	
Max. and min. transmission	0.97 and 0.78	
Refinement method	Full-matrix least-squares on F^2	
Data/restraints/parameters	74544 / 5815 / 1658	
Goodness-of-fit on F^2	0.90	
Final R indices (I>2sigma(I))	R1 = 0.087, wR2 = 0.193	
Largest diff. peak and hole	0.63 and -0.48 $eÅ^{-3}$	

Table S3. Crystal structure, crystal data and structure refinement of **3b** (CCDC 2208803).

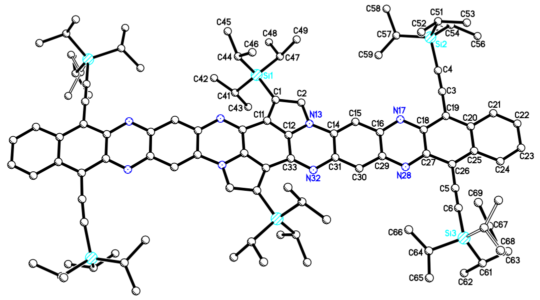

Identification code	mai26	
Empirical formula	$C_{104}H_{140}N_8Si_6$	
Formula weight	1670.77	
Temperature	200(2) K	
Wavelength	1.54178 Å	
Crystal system	monoclinic	
Space group	$P2_1/n$	
Z	2	
Unit cell dimensions	a = 16.0793(3) Å	α = 90 deg.
	b = 21.5044(5) Å	β =116.088(1) deg.
	c = 16.7375(3) Å	γ = 90 deg.
Volume	5197.79(19) $Å^3$	
Density (calculated)	1.07 g/cm^3	
Absorption coefficient	1.10 mm^{-1}	
Crystal shape	rhombohedral	
Crystal size	0.120 x 0.074 x 0.035 mm^3	
Crystal colour	dark blue	
Theta range for data collection	3.2 to 71.6 deg.	
Index ranges	$-13\leq h\leq 19$, $-22\leq k\leq 26$, $-20\leq l\leq 18$	
Reflections collected	32930	
Independent reflections	9762 (R(int) = 0.0297)	
Observed reflections	6977 ($I > 2\sigma(I)$)	
Absorption correction	Semi-empirical from equivalents	
Max. and min. transmission	1.75 and 0.54	
Refinement method	Full-matrix least-squares on F^2	
Data/restraints/parameters	9762 / 294 / 580	
Goodness-of-fit on F^2	1.04	
Final R indices (I>2sigma(I))	R1 = 0.062, wR2 = 0.166	
Largest diff. peak and hole	0.78 and -0.44 $eÅ^{-3}$	

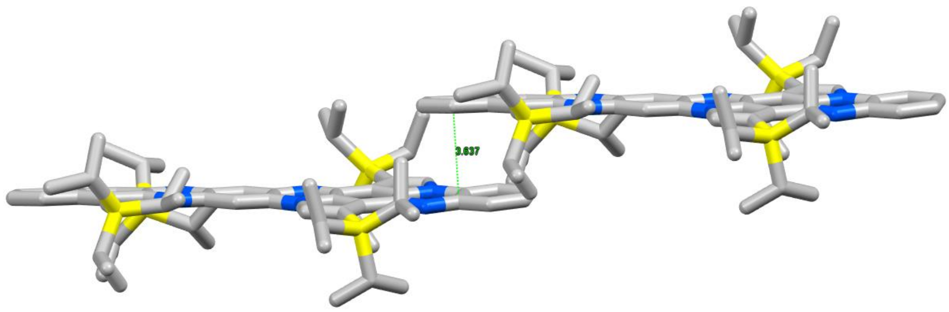

Figure S15. Estimation of π-π-distance by measuring the distance between the two atoms indicated as mean planes through the corresponding arene rings are not parallel in the crystal structure of **2b**.

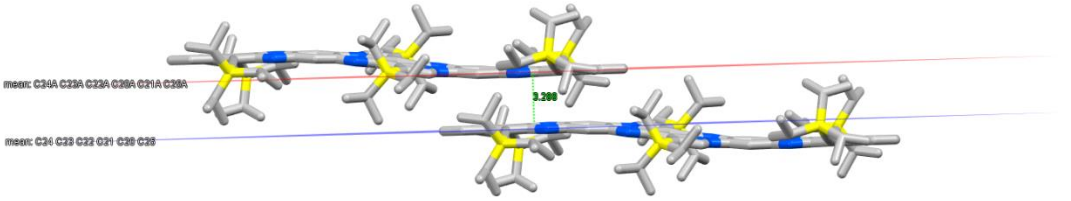

Figure S16. Estimation of π-π-distance in the crystal structure of **3b** by measuring the distance between two planes containing one benzene unit each.

6 Computational Details

6.1 Calculations of the Optoelectronic Properties

Calculations were performed using Gaussian 16.[4] TMS groups were used instead of TIPS groups to simplify calculations (inidicated by asterisk). First, the gas-phase ground-state equilibrium geometry of the molecules was optimized at the B3LYP/def2-SVP level of theory. Afterwards, the received geometries were refined using the B3LYP/def2-TZVP level of theory. FMO calculations were performed starting from the optimized geometries on the B3LYP/def2-TZVP level of theory.[4]

6.1.1 FMO Calculations

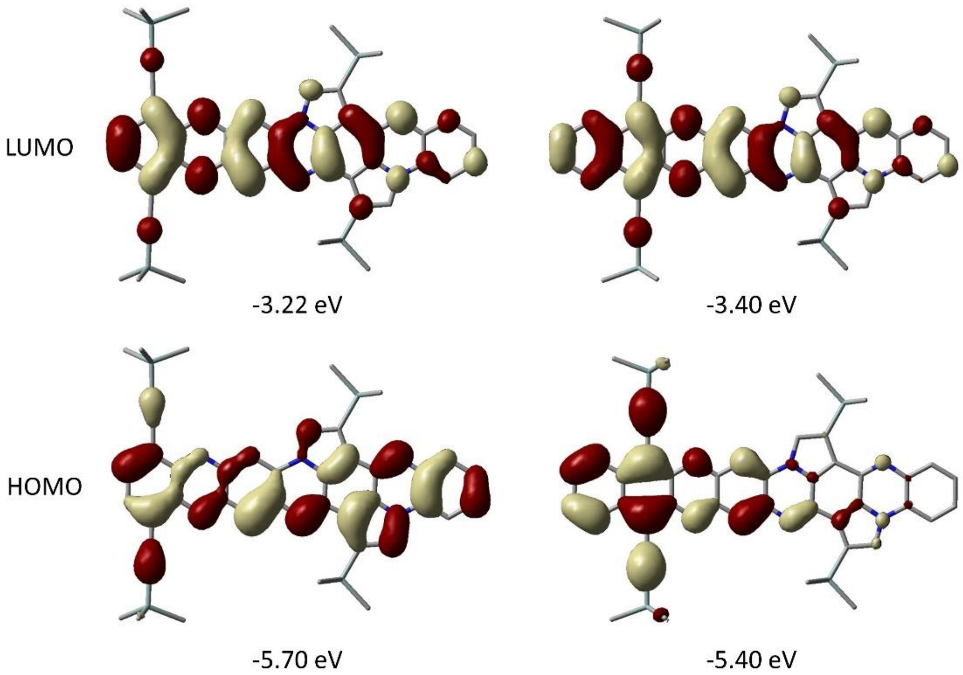

Figure S17. Calculated FMOs. Left: **2a***; Right: **2b***.

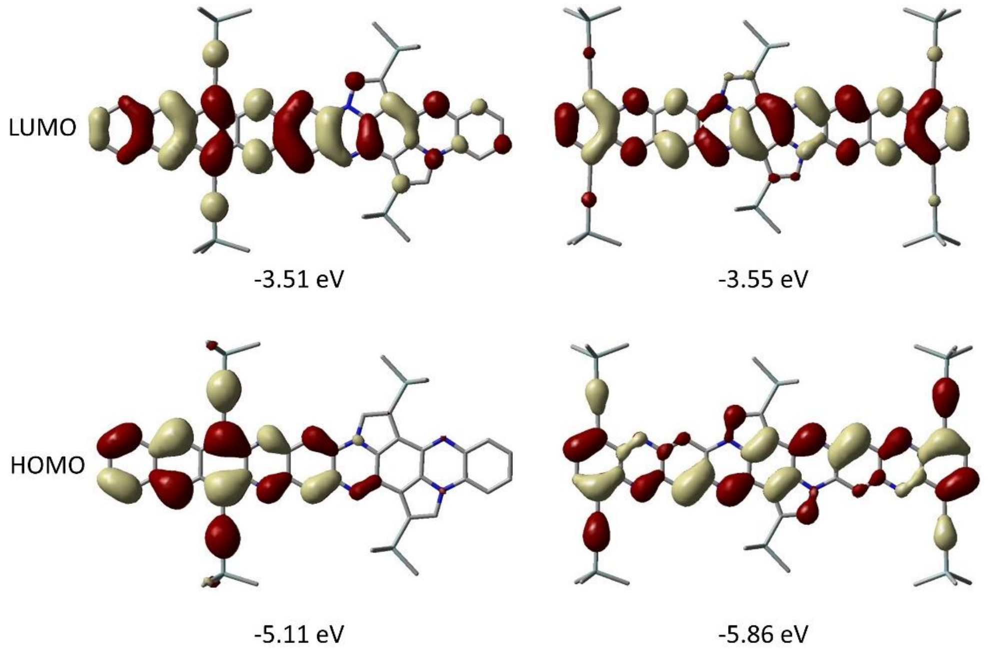

Figure S18. Calculated FMOs. Left: **2c***; Right: **3a***.

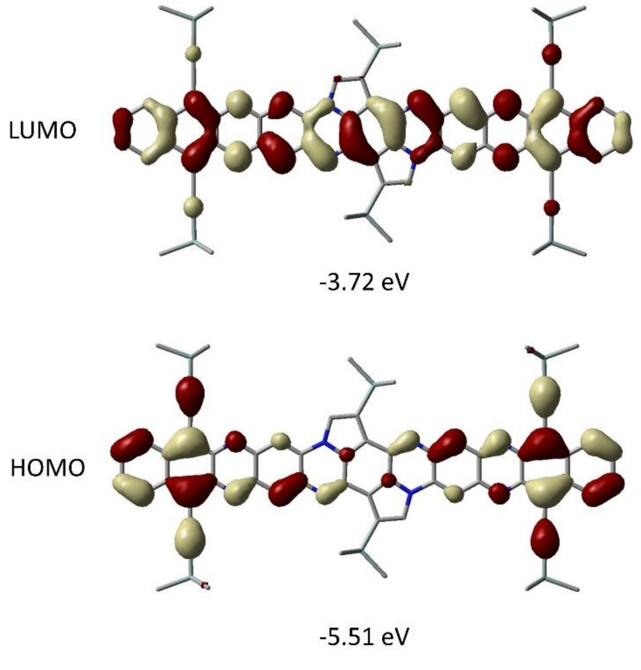

Figure S19. Calculated FMOs of **3b***.

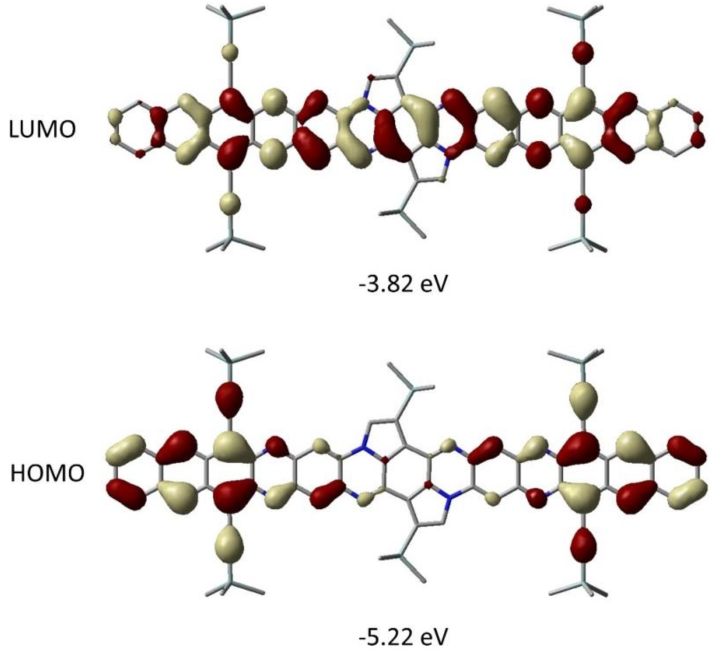

Figure S20. Calculated FMOs of **3c***.

6.1.2 Calculation of theoretical electron mobilities

Transfer integrals were calculated using the ADF program.[5] DZ basis set and GGA PW91 as functional were used. The calculation was performed for each dimer pair of a crystal structure. The used dimers and the corresponding transfer integrals are shown below. Reorganization energies were calculated using the four point method. Therefore, a geometry optimization of the isolated monomer in the gas phase was performed for the neutral ($E_{(M)}$) and the anionic species ($E^{-}_{(M-)}$) was performed. Afterwards single point energy calculations were perfomed starting from the coordinates of the neutral specie and charge the molecule negative ($E_{(M-)}$) and starting from the coordinates of the anionic specie where the charge was set neutral ($E^{-}_{(M)}$).[6] A first geometry optimization was performed using Gaussian 16 and the B3LYP/def2svp level of theory. A second Geometry optimization as well as single point calculations were performed using Gaussian 16 and the B3LYP/def2tzvp level of theory.

$$\lambda = \lambda_1 + \lambda_2$$
$$\lambda_1 = E_{(M-)} - E_{(M)}$$
$$\lambda_2 = E^{-}_{(M)} - E^{-}_{(M-)}$$

The reorganization energies and the transfer integrals were used to calculate the electron transfer rate using the Marcus theory.[7] The results for all possible transfer paths are summarized in the figures below.

$$k_{ET} = \frac{4\pi}{h}\frac{V^2}{\sqrt{4\pi k_b T}} e^{-\frac{\lambda}{4 k_b T}}$$

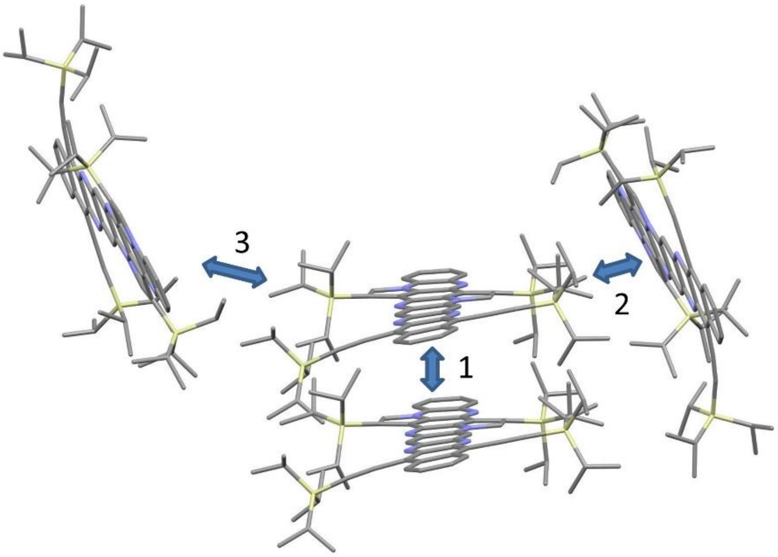

Dimer Pair	Transfer Integral V (for e$^-$) [meV]	Reorganisation Energy λ [meV]	Electron Transfer Rate k_{ET} [1/s]
1	19.2		4.93x10^{12}
2	1.1	160	1.58x10^{10}
3	1.1		1.58x10^{10}

Figure S21. Top: dimer pairs of **2b** used for the calculation of transfer integrals; bottom: calculated transfer integrals, reorganization energies and electron transfer rates.

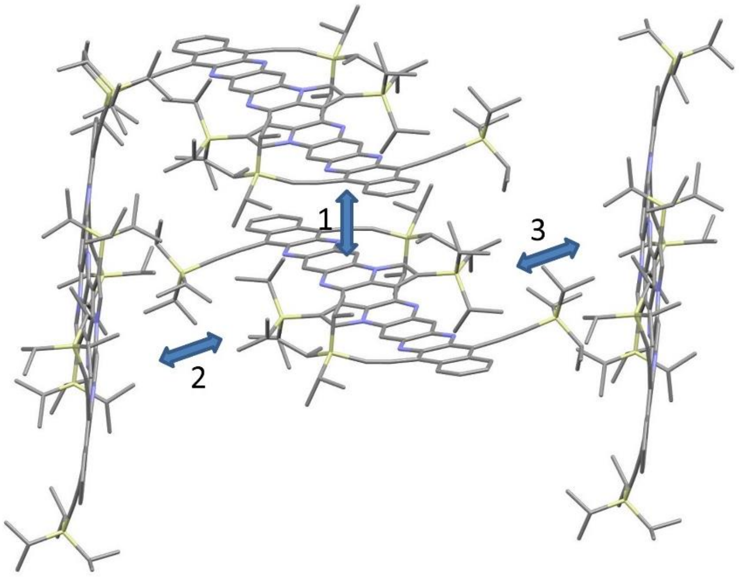

Dimer Pair	Transfer Integral V (for e^-) [meV]	Reorganisation Energy λ [meV]	Electron Transfer Rate k_{ET} [1/s]
1	30.4		1.31×10^{13}
2	2.2	143	6.91×10^{10}
3	0.3		9.57×10^{8}

Figure S22. Top: dimer pairs of **3b** used for the calculation of transfer integrals; bottom: calculated transfer integrals, reorganization energies and electron transfer rates.

Afterwards the theoretical electron mobility was calculated assuming a charge carrier diffusion in the crystal. The diffusion coefficient D was calculated as followed.[8]

$$D \approx \frac{1}{2n}\sum_i r_i^2 k_{ETi} P_i$$

$$P_i = \frac{k_{ETi}}{\sum_i k_{ETi}}$$

n: dimension; r_i: intermolecular distance between two molecules; P_i: hopping probability for path i.

The theoretical electron mobility was calculated using the following formular:[9]

$$\mu_{theo} = \frac{e}{k_b T} D$$

The results are shown in the manuscript.

6.1.3 TD-DFT calculations

The TD-DFT calculations were performed on the CAM-B3LYP/def2-TZVP level of theory as implemented in the Gaussian16[4] software package using the optimized gas phase structure of the ground state. Furthermore, a PCM model was used to describe the influence of the solvent used in the experiments, namely DCM. The first 20 excited singlet states were calculated. The absorption spectra were generated by apply a gauss function (half width 0.1 eV) on the transitions.

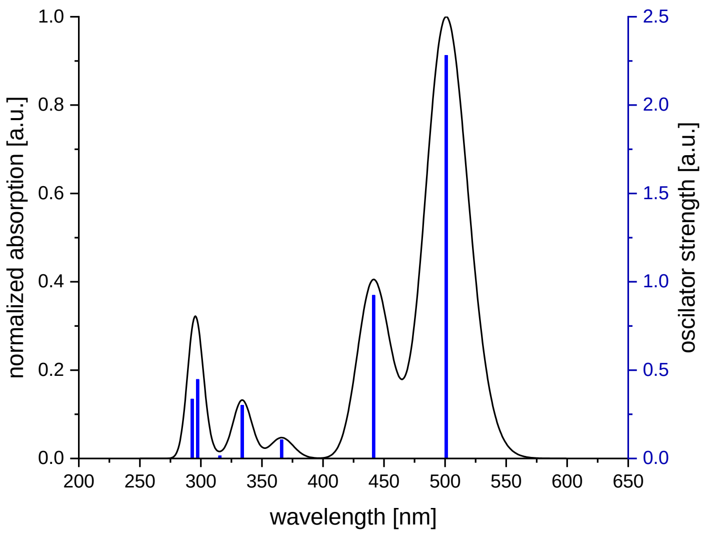

Figure S23. Calculated absorption spectra (black, half width 0.1 eV) and transitions (blue) obtained by TD-DFT (CAMB3LYP def2-TZVP) calculation with a PCM solvent model (DCM) of **3a**.

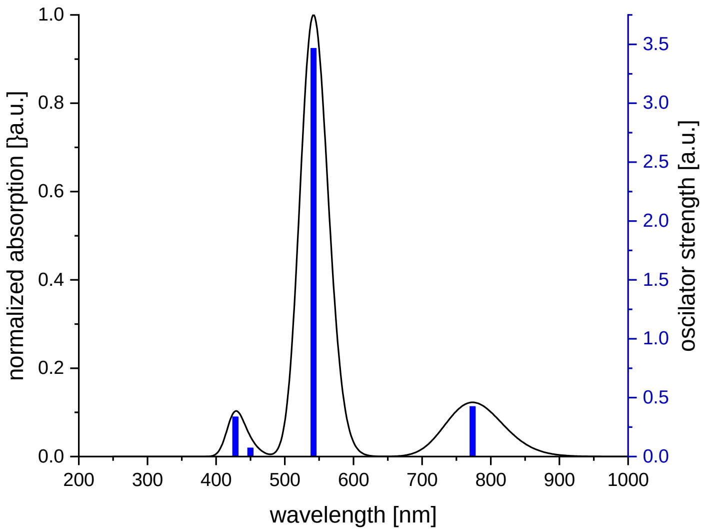

Figure S24. Calculated absorption spectra (black, half width 0.1 eV) and transitions (blue) obtained by TD-DFT (CAMB3LYP def2-TZVP) calculation with a PCM solvent model (DCM) of **3c**.

6.1.4 Coordinates of the Optimized Geometries

2a*:

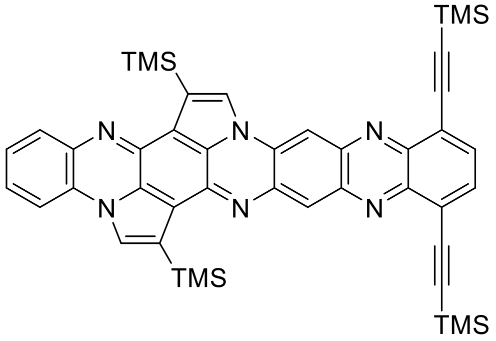

C	7.15088600	0.61240500	0.00090800
C	7.15514300	-0.80988500	0.00059700
N	5.91009900	-1.43400900	0.00011400
C	4.77488000	-0.66912700	0.00006400
C	4.85263700	0.75793600	0.00037300
N	6.00450500	1.37929200	0.00076000
C	3.66362500	-1.50514000	-0.00041900
C	2.36442400	-0.87081400	-0.00046600
C	2.44194100	0.56185100	-0.00024000
C	3.55135800	1.39589200	0.00016000
N	1.21543600	-1.49473600	-0.00062900
C	0.05977100	-0.73913300	-0.00052000
C	0.05348500	0.71747700	-0.00043800
N	1.30884100	1.32826600	-0.00039200
C	3.07174500	2.75255100	0.00024300
C	1.69324900	2.65541900	-0.00013500
C	4.14669700	-2.85851600	-0.00074700
C	5.52695400	-2.76130900	-0.00038400
C	-1.15510000	-1.39222000	-0.00050400
C	-2.37699800	-0.68624600	-0.00023200
C	-2.35858200	0.75462300	-0.00018600
C	-1.11384600	1.42896200	-0.00037600
C	8.39476400	1.26830100	0.00139400
C	9.57780000	0.55955000	0.00157600
C	9.55808900	-0.83916500	0.00126800
C	8.35526200	-1.52111100	0.00077900
N	-3.53517800	-1.35630200	0.00000900
C	-4.66330700	-0.65244500	0.00003800
C	-4.64228300	0.79114900	0.00007900
N	-3.49114700	1.46059800	0.00008800
C	-5.92889200	-1.34522400	-0.00007900
C	-7.08787300	-0.59768900	0.00005200
C	-7.06621000	0.80911500	0.00009000
C	-5.88467600	1.52135000	-0.00000100
C	-5.97589200	-2.76233300	-0.00052200
C	-6.04942100	-3.97350100	-0.00116700
C	-5.89014700	2.93968400	-0.00051000
C	-5.93076000	4.15242400	-0.00141900
Si	4.01257500	4.38679800	0.00080700
C	5.07136300	4.51704300	-1.54553700
C	5.07117200	4.51606600	1.54737900
C	2.73552800	5.77382400	0.00130300
Si	3.20495400	-4.49370000	-0.00207200
C	2.14996600	-4.62219600	-1.55019100
C	2.14841100	-4.62401800	1.54491600

C	4.48484700	-5.87935400	-0.00209800
Si	-6.11829700	-5.81112300	0.00089900
C	-4.42717600	-6.46910200	-0.48171200
C	-7.41860300	-6.36108100	-1.23950600
C	-6.58024500	-6.38714000	1.72908700
Si	-5.95871000	5.99064300	-0.00122400
C	-4.24303000	6.61050400	-0.44894300
C	-6.44211300	6.58011300	1.71642900
C	-7.21906200	6.56974100	-1.26903200
H	0.95518800	3.43775000	-0.00041100
H	6.26440200	-3.54403600	-0.00049900
H	-1.17552600	-2.47292400	-0.00089500
H	-1.12815300	2.50962600	-0.00067000
H	8.38697700	2.35006700	0.00162400
H	10.52336200	1.08578400	0.00195800
H	10.48653300	-1.39498900	0.00141400
H	8.34435000	-2.60244700	0.00055000
H	-8.04065200	-1.10963600	-0.00005400
H	-8.00281900	1.35011100	0.00000500
H	4.45971300	4.47175700	-2.44955300
H	5.61875500	5.46335700	-1.56011100
H	5.79658600	3.70317600	-1.58298300
H	4.45941200	4.47021300	2.45129500
H	5.61854500	5.46238200	1.56260900
H	5.79639800	3.70218800	1.58441800
H	2.09472800	5.74178500	-0.88307000
H	2.09514300	5.74125200	0.88597100
H	3.23938100	6.74380300	0.00148300
H	2.76299900	-4.57480400	-2.45319400
H	1.60307600	-5.56868600	-1.56704100
H	1.42377500	-3.80925300	-1.58683000
H	2.76044300	-4.57746900	2.44863700
H	1.60183000	-5.57072800	1.56008000
H	1.42203700	-3.81123800	1.58158600
H	3.98232900	-6.85000300	-0.00288600
H	5.12555800	-5.84570200	-0.88658100
H	5.12463400	-5.84662300	0.88309100
H	-3.66167100	-6.13332300	0.22061200
H	-4.42301300	-7.56224500	-0.48976000
H	-4.14023400	-6.12319000	-1.47675400
H	-8.40341200	-5.96721700	-0.97963400
H	-7.49004600	-7.45155500	-1.26838800
H	-7.17492100	-6.01378300	-2.24565000
H	-6.63027500	-7.47834800	1.77302900
H	-5.84462300	-6.05418600	2.46406000
H	-7.55369300	-5.99386500	2.02928100
H	-3.94239500	6.25490200	-1.43653600
H	-4.21458800	7.70321300	-0.46039800
H	-3.50074800	6.26126200	0.27162500
H	-7.43012400	6.20944800	1.99716400
H	-6.46837800	7.67222800	1.75814900
H	-5.72957600	6.23175500	2.46686700
H	-6.96233200	6.21621500	-2.26974000
H	-8.21787200	6.19864100	-1.02997300
H	-7.26496700	7.66153600	-1.29952500

2b*:

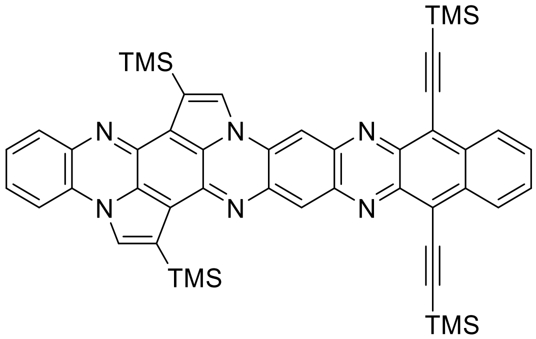

C	10.06030200	-0.80345200	-0.00016400
C	10.07405500	0.59545900	-0.00019200
C	8.88809700	1.29906300	-0.00016500
C	7.64700600	0.63766800	-0.00011000
C	7.65729700	-0.78476900	-0.00008300
C	8.86061900	-1.49072900	-0.00011100
N	6.49755000	1.39948100	-0.00008800
C	5.34825300	0.77314000	-0.00004200
C	5.27670900	-0.65406200	-0.00001200
N	6.41521800	-1.41408800	-0.00003300
C	4.04453900	1.40585000	-0.00000900
C	2.93858000	0.56753500	0.00002400
C	2.86733100	-0.86601700	0.00004700
C	4.16895700	-1.49464900	0.00003400
N	1.80281900	1.32921800	0.00000300
C	0.54878800	0.71523900	0.00002000
C	0.56153800	-0.74624400	0.00004800
N	1.72122500	-1.49495100	0.00006100
C	-0.61847600	1.42135400	0.00000900
C	-1.86569000	0.74301700	0.00001600
C	-1.87755500	-0.70458600	0.00003400
C	-0.64784800	-1.40400200	0.00005800
C	3.55979300	2.76120300	0.00001700
C	2.18215200	2.65825400	-0.00004800
C	4.65786300	-2.84612800	0.00005500
C	6.03758400	-2.74316500	-0.00000500
N	-2.99289600	1.44145000	0.00000400
C	-4.15287100	0.76675500	-0.00000900
C	-4.16705500	-0.67842000	-0.00000900
N	-3.02462500	-1.37747300	0.00002400
C	-5.37674100	1.49142000	-0.00001800
C	-6.60037900	0.79021400	-0.00005800
C	-6.61573300	-0.65165000	-0.00007100
C	-5.40797100	-1.37815600	-0.00003500
C	-7.84823800	1.48071200	-0.00008400
C	-9.03078500	0.80442500	-0.00012700
C	-9.04605800	-0.61534700	-0.00014900
C	-7.87815400	-1.31613600	-0.00012200
C	-5.40735700	-2.79344300	0.00000000
C	-5.34839500	2.90705500	0.00002300
C	-5.42874000	-4.00771800	0.00002300
C	-5.35004500	4.12151500	0.00005200
Si	3.72288800	-4.48534800	0.00011800
C	2.66782000	-4.61892300	1.54775200
C	2.66766400	-4.61894300	-1.54740800

C	5.00877300	-5.86539800	0.00006900
Si	4.49378500	4.39933200	0.00004500
C	5.55198000	4.53367100	-1.54635000
C	3.21058200	5.78068300	-0.00004500
C	5.55182400	4.53372600	1.54654100
Si	-5.39021000	-5.84555000	-0.00002700
C	-4.48741800	-6.42060700	1.54383800
C	-7.15811500	-6.48402800	-0.00108700
C	-4.48554500	-6.42047700	-1.54284300
Si	-5.29460100	5.95829400	0.00005700
C	-4.38571300	6.52692800	1.54314600
C	-4.38520600	6.52696500	-1.54271700
C	-7.05612600	6.61365300	-0.00026300
H	10.99118300	-1.35518300	-0.00018600
H	11.01737900	1.12566900	-0.00023500
H	8.87554700	2.38077600	-0.00018700
H	8.85449000	-2.57209100	-0.00009000
H	-0.63687600	2.50194000	0.00000000
H	-0.66324300	-2.48476700	0.00008000
H	1.44074700	3.43738700	-0.00008500
H	6.77836800	-3.52272900	-0.00002400
H	-7.83217200	2.56154400	-0.00007100
H	-9.96618200	1.34962100	-0.00014600
H	-9.99285600	-1.14044700	-0.00019200
H	-7.88428500	-2.39709100	-0.00014400
H	2.12561500	-5.56812900	1.56424500
H	3.28006300	-4.56873600	2.45112900
H	1.93774600	-3.80944900	1.58407900
H	2.12545200	-5.56814500	-1.56383700
H	3.27981800	-4.56876700	-2.45084600
H	1.93759300	-3.80946300	-1.58366900
H	5.64891900	-5.82941200	0.88487000
H	4.51051400	-6.83823700	0.00013900
H	5.64879100	-5.82947100	-0.88482600
H	6.09518000	5.48238900	-1.56128900
H	6.28083300	3.72304100	-1.58357600
H	4.94056000	4.48533800	-2.45036100
H	3.71007300	6.75290600	-0.00003100
H	2.57018300	5.74544300	0.88451000
H	2.57028100	5.74541900	-0.88467000
H	4.94031200	4.48542400	2.45049100
H	6.28066700	3.72309300	1.58386600
H	6.09502500	5.48244300	1.56150400
H	-4.43226100	-7.51195700	1.57721000
H	-3.46828300	-6.02983800	1.56935600
H	-4.99695200	-6.08140100	2.44801900
H	-7.17547500	-7.57703700	-0.00111400
H	-7.70141400	-6.14120100	-0.88423400
H	-7.70243200	-6.14123900	0.88144800
H	-3.46629100	-6.02993100	-1.56696200
H	-4.43059300	-7.51183200	-1.57637900
H	-4.99383900	-6.08096300	-2.44760500
H	-4.32197500	7.61779200	1.57649700
H	-4.89705700	6.19169000	2.44777600
H	-3.36947700	6.12851300	1.56827900
H	-4.32123900	7.61782200	-1.57588200

H	-4.89638000	6.19197500	-2.44753600
H	-3.36904400	6.12835100	-1.56765200
H	-7.60325400	6.27601600	0.88250400
H	-7.06321800	7.70676800	-0.00041500
H	-7.60297500	6.27577500	-0.88311200

2c*:

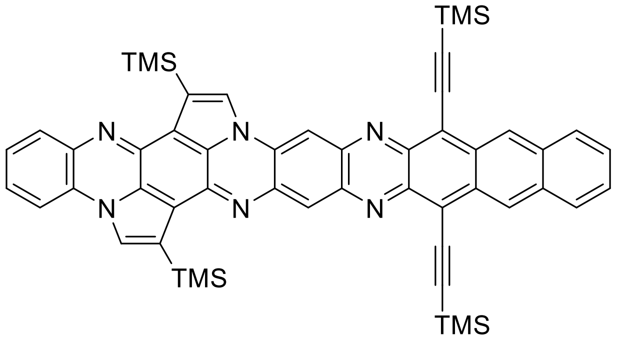

C	-8.26859800	0.65332900	-0.00019300
C	-8.28261600	-0.76919300	-0.00124800
N	-7.04238700	-1.40168300	-0.00168800
C	-5.90186600	-0.64465600	-0.00107200
C	-5.96954900	0.78260000	-0.00005400
N	-7.11728900	1.41200400	0.00037500
C	-4.79629600	-1.48811100	-0.00168100
C	-3.49329100	-0.86292900	-0.00114500
C	-3.56060400	0.57103400	-0.00019100
C	-4.66434300	1.41209700	0.00037600
N	-2.34885900	-1.49502300	-0.00143000
C	-1.18695600	-0.75053600	-0.00085300
C	-1.17014300	0.71347500	-0.00007300
N	-2.42307700	1.32975600	0.00031100
C	-4.17626600	2.76658500	0.00127300
C	-2.79913400	2.66002800	0.00119200
C	-5.28879000	-2.83843800	-0.00275800
C	-6.66813900	-2.73186900	-0.00272100
C	0.01945700	-1.41149000	-0.00104700
C	1.25312900	-0.71609500	-0.00058400
C	1.24534700	0.73520500	-0.00014300
C	-0.00247400	1.41634800	0.00020300
C	-9.50796900	1.31810900	0.00027400
C	-10.69570600	0.61767100	-0.00027700
C	-10.68562900	-0.78135700	-0.00132200
C	-9.48790000	-1.47191300	-0.00180600
N	2.39471200	-1.39153100	-0.00059800
C	3.54413400	-0.69613800	-0.00054300
C	3.53410700	0.75257700	-0.00042100
N	2.37041200	1.42989000	-0.00007100
C	4.77434400	-1.40030800	-0.00066900
C	5.99673200	-0.67808100	-0.00117000
C	5.98542100	0.77132500	-0.00112800
C	4.75074800	1.47458700	-0.00061500
C	7.23660800	-1.34165700	-0.00176100
C	8.44059200	-0.65703800	-0.00226600
C	8.42916100	0.78732300	-0.00212900
C	7.21432200	1.45344100	-0.00156000
C	9.70202400	-1.33079400	-0.00293500

C	10.86770600	-0.62979400	-0.00338500
C	10.85634400	0.79756500	-0.00320500
C	9.67977800	1.48041500	-0.00261000
C	4.76851200	-2.81408200	0.00004800
C	4.78958000	-4.02885600	0.00109200
C	4.72442300	2.88882200	-0.00031800
C	4.73014400	4.10373100	0.00002700
Si	-5.10623600	4.40708000	0.00235900
C	-6.16412100	4.54268900	1.54890300
C	-6.16425700	4.54465400	-1.54391800
C	-3.82001700	5.78536700	0.00317700
Si	-4.35774000	-4.48010800	-0.00441200
C	-3.30287900	-4.61743500	1.54295500
C	-3.30357100	-4.61452000	-1.55246400
C	-5.64730200	-5.85672200	-0.00534800
Si	4.75306000	-5.86651100	0.00620900
C	2.97006200	-6.42590300	-0.17618000
C	5.46967000	-6.47423800	1.63429900
C	5.78892700	-6.49099300	-1.43279100
Si	4.67772800	5.94023500	0.00076600
C	2.87941800	6.48301200	0.00060200
C	5.55175600	6.56731300	-1.54067000
C	5.55107000	6.56601400	1.54312000
H	-2.05563900	3.43719700	0.00171400
H	-7.41096300	-3.50949300	-0.00338200
H	0.03182600	-2.49229600	-0.00174600
H	0.01883600	2.49687600	0.00064000
H	-9.49249900	2.39977800	0.00107600
H	-11.63765000	1.15031900	0.00009600
H	-11.61800900	-1.33055300	-0.00175700
H	-9.48471400	-2.55328100	-0.00261100
H	7.24071700	-2.42349800	-0.00185400
H	7.20226300	2.53521500	-0.00149000
H	9.70881100	-2.41413600	-0.00313200
H	11.81652100	-1.15130900	-0.00391300
H	11.79681600	1.33401500	-0.00357100
H	9.66991400	2.56373700	-0.00249600
H	-5.55276400	4.49229000	2.45284600
H	-6.70501100	5.49271800	1.56454400
H	-6.89496300	3.73383000	1.58567400
H	-5.55297600	4.49542900	-2.44797700
H	-6.70516600	5.49469200	-1.55829200
H	-6.89508400	3.73582800	-1.58166500
H	-3.17957800	5.74824700	0.88760400
H	-3.17966900	5.74937500	-0.88136200
H	-4.31738700	6.75868200	0.00382400
H	-3.91493600	-4.56705400	2.44645500
H	-2.76253000	-5.56770500	1.55812200
H	-2.57111000	-3.80953400	1.58000700
H	-3.91608000	-4.56245100	-2.45556300
H	-2.76313200	-5.56469400	-1.56972300
H	-2.57182100	-3.80655100	-1.58838400
H	-5.15155400	-6.83083900	-0.00633600
H	-6.28708300	-5.81990600	0.87968800
H	-6.28747000	-5.81832900	-0.89003700
H	2.53797200	-6.06075500	-1.10997100

H	2.90302100	-7.51703800	-0.17609600
H	2.35588200	-6.04927100	0.64411700
H	6.50141300	-6.14008900	1.76211300
H	5.46284100	-7.56666700	1.67636400
H	4.89109000	-6.10132800	2.48185500
H	5.78822400	-7.58377000	-1.46456400
H	5.39917700	-6.12682500	-2.38551700
H	6.82587500	-6.15917700	-1.34787800
H	2.35722200	6.10687300	0.88263100
H	2.80167200	7.57339000	0.00105400
H	2.35760000	6.10763100	-0.88197300
H	6.59467600	6.24431300	-1.56318400
H	5.53814500	7.65988100	-1.57641700
H	5.06819200	6.19507000	-2.44610400
H	5.06709700	6.19300600	2.44802100
H	6.59397700	6.24298600	1.56583100
H	5.53744500	7.65855100	1.57978500

3a*:

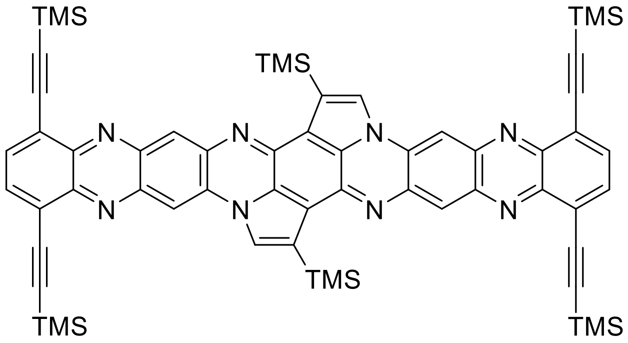

C	3.53926400	0.72129000	-0.00319900
C	3.56169900	-0.73457000	-0.00389800
N	2.31399500	-1.35903100	-0.00484400
C	1.17341400	-0.60528000	-0.00480200
C	1.23443200	0.82749200	-0.00458900
N	2.37508300	1.46410300	-0.00387800
C	0.07263400	-1.45053400	-0.00490000
C	-1.23403800	-0.82760200	-0.00455800
C	-1.17301500	0.60517200	-0.00486600
C	-0.07223600	1.45042400	-0.00501200
N	-2.37469600	-1.46420400	-0.00385600
C	-3.53888200	-0.72139300	-0.00327700
C	-3.56129800	0.73446800	-0.00406000
N	-2.31359200	1.35892200	-0.00499900
C	-0.56584700	2.80266000	-0.00537900
C	-1.94273300	2.69103500	-0.00538700
C	0.56624400	-2.80277000	-0.00512600
C	1.94313100	-2.69114400	-0.00512800
C	-4.74534000	-1.39049800	-0.00174600
C	-5.97478000	-0.69872700	-0.00080400
C	-5.97316200	0.74178100	-0.00200200
C	-4.73784500	1.43184800	-0.00368900
C	4.74570900	1.39040200	-0.00165400
C	5.97516800	0.69868000	-0.00058900
C	5.97357500	-0.74182400	-0.00175800
C	4.73826600	-1.43191900	-0.00346100
N	-7.12496000	-1.38342400	0.00133900

C	-8.26088500	-0.69409000	0.00204200
C	-8.25718900	0.75080900	0.00043600
N	-7.11529400	1.43393400	-0.00138400
C	-9.51814900	-1.40290100	0.00447800
C	-10.68536000	-0.66914200	0.00489700
C	-10.68070000	0.73841500	0.00303600
C	-9.50895700	1.46588800	0.00081000
N	7.12532500	1.38341400	0.00162800
C	8.26127700	0.69412400	0.00232000
C	8.25760700	-0.75078100	0.00071000
N	7.11572800	-1.43393900	-0.00109900
C	9.51854100	1.40295400	0.00466300
C	10.68576100	0.66920700	0.00518800
C	10.68112000	-0.73834800	0.00333800
C	9.50938700	-1.46584000	0.00103000
C	9.54761500	2.82036800	0.00632900
C	9.53220400	-2.88384700	-0.00135500
C	9.58725900	-4.09597800	-0.00394400
C	9.60560400	4.03235200	0.00774000
C	-9.54723800	-2.82031200	0.00662900
C	-9.60536200	-4.03229100	0.00878500
C	-9.53175000	2.88389600	-0.00128400
C	-9.58677300	4.09602800	-0.00349600
Si	-0.35844400	-4.44865600	-0.00563900
C	-1.41289600	-4.58698500	-1.55333000
C	-1.41307900	-4.58795700	1.54188700
C	0.93636600	-5.81886200	-0.00579200
Si	0.35879700	4.44855500	-0.00626200
C	1.41299200	4.58668100	-1.55412700
C	1.41370300	4.58812900	1.54108700
C	-0.93602700	5.81874600	-0.00631500
Si	9.63852500	-5.93451700	-0.00669800
C	10.90927400	-6.49408000	-1.27253700
C	7.93161100	-6.57393700	-0.45983000
C	10.12494500	-6.51929800	1.71145300
Si	9.65413300	5.87148100	0.01332900
C	10.94315700	6.43687400	-1.23149900
C	10.11664000	6.44746800	1.74112200
C	7.95355400	6.51045900	-0.46102800
Si	-9.65584400	-5.87136400	0.01446100
C	-7.89978400	-6.51307800	-0.15717200
C	-10.71179300	-6.44187700	-1.43138900
C	-10.41016600	-6.43936000	1.63910900
Si	-9.63796000	5.93457100	-0.00552500
C	-7.93113700	6.57410500	-0.45883500
C	-10.12389400	6.51866200	1.71300000
C	-10.90902600	6.49470800	-1.27078900
H	-2.68920400	3.46531400	-0.00583400
H	2.68960100	-3.46542500	-0.00547900
H	-4.75229100	-2.47136700	-0.00117500
H	-4.76553100	2.51221500	-0.00469900
H	4.75260400	2.47126600	-0.00129800
H	4.76597600	-2.51228500	-0.00443700
H	-11.63211700	-1.19209000	0.00669900
H	-11.62416100	1.26737700	0.00331000
H	11.63251200	1.19217100	0.00691600

H	11.62458900	-1.26729700	0.00359000
H	-0.80074300	-4.53253000	-2.45649900
H	-1.94865600	-5.53979800	-1.57035300
H	-2.14887700	-3.78291500	-1.59030800
H	-0.80107600	-4.53393500	2.44518500
H	-1.94864900	-5.54089600	1.55825800
H	-2.14914800	-3.78398800	1.57919300
H	0.44430800	-6.79484400	-0.00630300
H	1.57667900	-5.77874800	-0.89020000
H	1.57599200	-5.77933300	0.87915200
H	0.80067000	4.53205200	-2.45717100
H	1.94874000	5.53949400	-1.57147700
H	2.14899300	3.78263200	-1.59114600
H	0.80189100	4.53408000	2.44451100
H	1.94907800	5.54118700	1.55729800
H	2.14989400	3.78426900	1.57832000
H	-1.57649900	5.77853000	-0.89060200
H	-1.57549400	5.77929700	0.87874900
H	-0.44399000	6.79473800	-0.00702700
H	11.90262500	-6.11068400	-1.03021700
H	10.96935400	-7.58510900	-1.30509600
H	10.65044300	-6.14196600	-2.27319000
H	7.18341000	-6.23574900	0.25988900
H	7.91670200	-7.66686400	-0.47385100
H	7.62892000	-6.21995800	-1.44737400
H	11.10764500	-6.13687700	1.99499500
H	9.40632800	-6.18078800	2.46058200
H	10.16461700	-7.61103700	1.75195200
H	10.69906400	6.08874500	-2.23724200
H	11.00268200	7.52808500	-1.25879000
H	11.93324000	6.05334200	-0.97632600
H	9.38756600	6.10492600	2.47819700
H	10.15512200	7.53901900	1.78761400
H	11.09547600	6.06404100	2.03643800
H	7.93708100	7.60347500	-0.46741900
H	7.66627300	6.16297200	-1.45541900
H	7.19490300	6.16532300	0.24419700
H	-7.27321800	-6.16566900	0.66669800
H	-7.88383100	-7.60611600	-0.15642100
H	-7.44547300	-6.16952700	-1.08864700
H	-11.73047100	-6.05648900	-1.35258800
H	-10.76715000	-7.53319600	-1.46339600
H	-10.29735800	-6.09856700	-2.38145600
H	-10.45839100	-7.53062600	1.68282300
H	-9.81835100	-6.09470900	2.48950400
H	-11.42451700	-6.05346200	1.75977600
H	-7.62870800	6.22051500	-1.44659900
H	-7.91618600	7.66703600	-0.47241400
H	-7.18277000	6.23559200	0.26055900
H	-11.10653600	6.13616800	1.99664700
H	-10.16350300	7.61038500	1.75396700
H	-9.40509200	6.17980400	2.46179600
H	-10.65048400	6.14300000	-2.27165900
H	-11.90232700	6.11125500	-1.02835500
H	-10.96906900	7.58575400	-1.30287800

3b*:

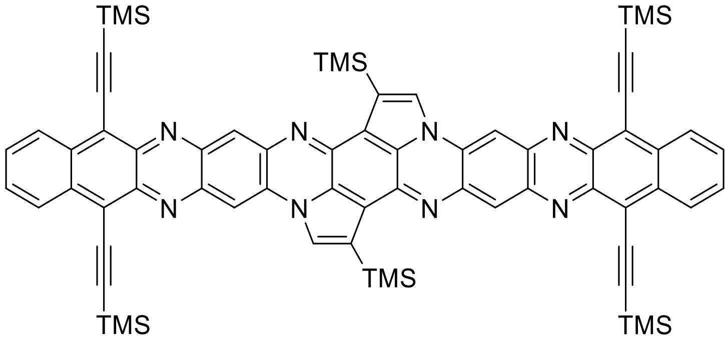

C	13.14230500	-0.73323100	0.00022800
C	13.14445000	0.68696400	0.00023300
C	11.97039600	1.37681600	0.00016700
C	10.71401800	0.70041800	0.00008600
C	10.71197600	-0.74208800	0.00006400
C	11.96652600	-1.42075600	0.00014400
C	9.50039900	1.41590500	0.00003200
C	8.26568500	0.70384800	-0.00002700
C	8.26463900	-0.74236600	-0.00005100
C	9.49586100	-1.45546600	-0.00003000
N	7.11768600	1.39159900	-0.00003900
C	5.97663400	0.70749400	-0.00005900
C	5.97728000	-0.73955200	-0.00007000
N	7.11193700	-1.42746900	-0.00007000
C	4.74162100	1.39657100	-0.00006600
C	3.53864400	0.72649600	-0.00007200
C	3.56301400	-0.73416900	-0.00008000
C	4.73729400	-1.42976900	-0.00008000
N	2.37302500	1.46635100	-0.00006800
C	1.23323200	0.82841200	-0.00004200
C	1.17387900	-0.60481600	-0.00004000
N	2.31456700	-1.35772000	-0.00009500
C	-0.07365000	1.45041900	-0.00001100
C	-1.17390300	0.60479700	0.00001300
C	-1.23325700	-0.82843100	-0.00003100
C	0.07362600	-1.45043900	-0.00006000
N	-2.31459000	1.35770200	0.00001300
C	-3.56303700	0.73415200	0.00003300
C	-3.53866900	-0.72651300	-0.00000100
N	-2.37305000	-1.46636900	-0.00004700
C	-4.73731400	1.42975500	0.00006000
C	-5.97730100	0.73954100	0.00004300
C	-5.97666000	-0.70750500	0.00003000
C	-4.74164900	-1.39658500	0.00000100
N	-7.11195200	1.42746500	0.00002300
C	-8.26465600	0.74236800	-0.00000100
C	-8.26571000	-0.70384600	0.00003100
N	-7.11771600	-1.39160400	0.00003700
C	-9.49587300	1.45547600	-0.00007100
C	-10.71199100	0.74210700	-0.00003700
C	-10.71404400	-0.70039900	0.00003600
C	-9.50043000	-1.41589500	0.00005600
C	-11.96653500	1.42078700	-0.00005900
C	-13.14232000	0.73327200	-0.00001000
C	-13.14447600	-0.68692300	0.00005900

C	-11.97042900	-1.37678500	0.00008200
C	0.56779300	-2.80300300	-0.00013500
C	1.94421000	-2.69031700	-0.00014900
C	-0.56781600	2.80298300	-0.00002800
C	-1.94423300	2.69029800	-0.00000800
C	9.48097700	-2.87117600	-0.00004600
C	9.49385700	-4.08553600	-0.00006000
C	9.48600500	2.83092500	0.00004000
C	9.49534000	4.04533600	0.00005600
C	-9.48097700	2.87118500	-0.00012300
C	-9.49386300	4.08554500	-0.00015300
C	-9.48604500	-2.83091500	0.00009800
C	-9.49537100	-4.04532600	0.00015200
Si	-0.35552400	-4.44950400	-0.00022200
C	-1.41002100	-4.58958700	-1.54776200
C	-1.41003700	-4.58975600	1.54729200
C	0.94077900	-5.81837400	-0.00028500
Si	0.35550100	4.44948500	-0.00007700
C	1.40995700	4.58962500	-1.54764000
C	1.41005300	4.58968300	1.54741500
C	-0.94080400	5.81835400	-0.00004900
Si	9.45748100	-5.92364100	-0.00009800
C	11.22594600	-6.55935200	0.00078300
C	8.55496600	-6.50026300	-1.54372500
C	8.55343800	-6.50025400	1.54263800
Si	9.44179400	5.88375000	0.00017800
C	11.20468100	6.53511500	-0.00118200
C	8.53446600	6.44964700	1.54452200
C	8.53202200	6.44978200	-1.54267500
Si	-9.45742400	5.92364700	-0.00010200
C	-8.55671700	6.50020100	1.54461200
C	-11.22586300	6.55941300	-0.00313200
C	-8.55151400	6.50026100	-1.54174400
Si	-9.44176300	-5.88373900	0.00028100
C	-8.53238100	-6.44975800	-1.54280700
C	-11.20462900	-6.53515900	-0.00060500
C	-8.53400500	-6.44959100	1.54438900
H	14.08283700	-1.26946800	0.00029500
H	14.08640500	1.22061800	0.00029200
H	11.96633900	2.45775300	0.00017700
H	11.96069200	-2.50165700	0.00013900
H	4.74682800	2.47742300	-0.00006000
H	4.76565200	-2.51010400	-0.00008000
H	-4.76567000	2.51009000	0.00008800
H	-4.74685800	-2.47743800	-0.00002300
H	-11.96069300	2.50168800	-0.00011800
H	-14.08284800	1.26951800	-0.00002200
H	-14.08643600	-1.22056900	0.00009300
H	-11.96638100	-2.45772200	0.00013700
H	2.69131300	-3.46396500	-0.00019600
H	-2.69133600	3.46394600	-0.00001200
H	-0.79824800	-4.53403900	-2.45111500
H	-1.94399700	-5.54342400	-1.56482400
H	-2.14739200	-3.78678300	-1.58466500
H	-0.79827000	-4.53433600	2.45065600
H	-1.94403500	-5.54358300	1.56423000

H	-2.14738900	-3.78693800	1.58429100
H	1.58062500	-5.77773600	-0.88502300
H	1.58060700	-5.77782800	0.88447000
H	0.44982100	-6.79490700	-0.00034100
H	0.79816200	4.53409700	-2.45097900
H	1.94392600	5.54346600	-1.56468900
H	2.14733100	3.78682500	-1.58458300
H	0.79830800	4.53423100	2.45079200
H	1.94404800	5.54351100	1.56437200
H	2.14740900	3.78686700	1.58436900
H	-1.58070300	5.77773000	-0.88474800
H	-1.58057900	5.77779200	0.88474500
H	-0.44984600	6.79488700	-0.00011800
H	11.76891700	-6.21547800	0.88370100
H	11.24478300	-7.65231300	0.00127100
H	11.76960000	-6.21625000	-0.88201300
H	7.53435600	-6.11318100	-1.56863200
H	8.50320800	-7.59171500	-1.57795400
H	9.06268100	-6.15883300	-2.44806900
H	9.06096000	-6.15981200	2.44746900
H	7.53321100	-6.11214400	1.56715100
H	8.50056400	-7.59166900	1.57626500
H	11.75038700	6.19664500	-0.88451200
H	11.21366400	7.62820400	-0.00113000
H	11.75177500	6.19655400	0.88125600
H	7.51848400	6.05075900	1.56995300
H	8.47042300	7.54047000	1.57856900
H	9.04689700	6.11420100	2.44845300
H	9.04299500	6.11436400	-2.44744100
H	7.51598500	6.05093800	-1.56650800
H	8.46797600	7.54061000	-1.57655700
H	-7.53639200	6.11247100	1.57106700
H	-8.50432300	7.59162400	1.57864800
H	-9.06592600	6.15930400	2.44832200
H	-11.76771200	6.21581400	-0.88684300
H	-11.24466100	7.65237500	-0.00332300
H	-11.77065200	6.21608100	0.87887700
H	-8.49845100	7.59167700	-1.57514400
H	-7.53130600	6.11202900	-1.56508600
H	-9.05799500	6.16002200	-2.44723100
H	-7.51635400	-6.05090200	-1.56690000
H	-8.46832900	-7.54058500	-1.57670400
H	-9.04358800	-6.11434700	-2.44744400
H	-11.75151500	-6.19656500	0.88194900
H	-11.21357800	-7.62824900	-0.00048800
H	-11.75056500	-6.19675800	-0.88381900
H	-8.46989900	-7.54041000	1.57842200
H	-7.51803700	-6.05065000	1.56955400
H	-9.04621700	-6.11416800	2.44845200

3c*:

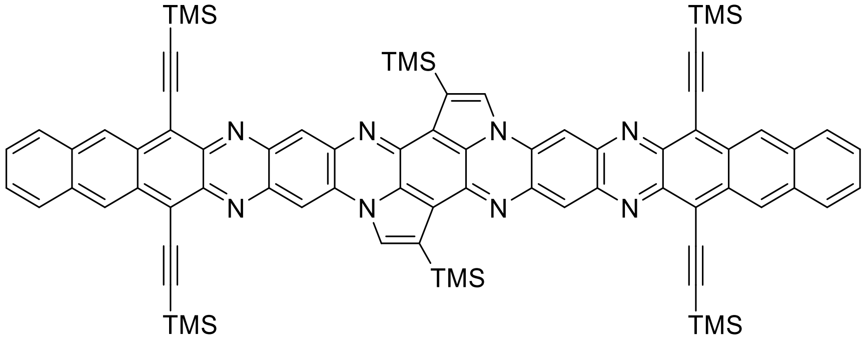

C	3.55243200	0.73586600	-0.00052100
C	3.57704500	-0.73519400	-0.00056600
N	2.32585500	-1.36167500	-0.00056300
C	1.18083800	-0.60652500	-0.00051800
C	1.24005400	0.83370100	-0.00070800
N	2.38457300	1.47204800	-0.00068100
C	0.07381100	-1.45644100	-0.00038400
C	-1.24018400	-0.83332300	-0.00040000
C	-1.18098400	0.60690300	-0.00075600
C	-0.07395500	1.45682100	-0.00097400
N	-2.38468400	-1.47168700	-0.00020900
C	-3.55255000	-0.73553200	-0.00034700
C	-3.57720800	0.73553100	-0.00085900
N	-2.32601800	1.36203400	-0.00112200
C	-0.57141000	2.81229200	-0.00158500
C	-1.95649700	2.69763000	-0.00166900
C	0.57123900	-2.81191700	-0.00039600
C	1.95632600	-2.69726700	-0.00053400
C	-4.76422600	-1.40746300	-0.00008200
C	-6.00793000	-0.71595200	-0.00029300
C	-6.00825900	0.74244600	-0.00080000
C	-4.75866800	1.43416800	-0.00109700
C	4.76414700	1.40773800	-0.00043700
C	6.00784500	0.71620200	-0.00045500
C	6.00808200	-0.74219700	-0.00066500
C	4.75847300	-1.43386800	-0.00067900
N	-7.15053700	-1.39810700	-0.00006100
C	-8.30621300	-0.71120300	-0.00072800
C	-8.30503100	0.74580700	-0.00126900
N	-7.14358500	1.42919100	-0.00103600
C	-9.53951600	-1.42853500	-0.00092500
C	-10.77205100	-0.70888600	-0.00213200
C	-10.76985600	0.74757000	-0.00278100
C	-9.53408500	1.46438700	-0.00209000
C	-12.01412700	-1.38403600	-0.00277100
C	-13.23008100	-0.70405500	-0.00409800
C	-13.22781900	0.74771800	-0.00480700
C	-12.00968600	1.42496800	-0.00409900
C	-14.49233700	-1.38964800	-0.00484300
C	-15.67034500	-0.69178000	-0.00619700
C	-15.66805700	0.74153400	-0.00692900
C	-14.48806100	1.43637700	-0.00626600
C	-9.51863700	-2.84818600	0.00019700
C	-9.52478000	-4.07407900	0.00153100
C	-9.51190300	2.88478900	-0.00213200
C	-9.51975700	4.11059800	-0.00186000

N	7.15050500	1.39828400	-0.00037000
C	8.30614200	0.71128500	-0.00114400
C	8.30483900	-0.74573400	-0.00146800
N	7.14334900	-1.42902400	-0.00089400
C	9.53954000	1.42845600	-0.00166700
C	10.77199900	0.70865200	-0.00318800
C	10.76965400	-0.74779900	-0.00357100
C	9.53380400	-1.46446100	-0.00241500
C	12.01415700	1.38365700	-0.00438700
C	13.23003300	0.70352800	-0.00599700
C	13.22761100	-0.74824300	-0.00637900
C	12.00940000	-1.42534900	-0.00513000
C	14.49236400	1.38898300	-0.00736900
C	15.67029400	0.69098100	-0.00896800
C	15.66784600	-0.74233300	-0.00934100
C	14.48777400	-1.43704500	-0.00810600
C	9.51897800	2.84810400	-0.00037000
C	9.52576000	4.07398900	0.00139200
C	9.51144000	-2.88486100	-0.00212800
C	9.51912400	-4.11067100	-0.00152900
Si	0.36661200	4.46126800	-0.00268100
C	1.42908000	4.59013900	-1.55668200
C	1.43022000	4.59169800	1.55049100
C	-0.93204800	5.84062200	-0.00269300
Si	-0.36682500	-4.46088100	-0.00055600
C	-1.42944500	-4.59054100	-1.55439900
C	-1.43027300	-4.59049100	1.55277000
C	0.93182800	-5.84024500	-0.00003700
Si	-9.46009200	-5.92333600	0.00648800
C	-7.66122300	-6.45562500	-0.19622200
C	-10.50718700	-6.56647000	-1.42830100
C	-10.15305500	-6.54364600	1.65073900
Si	-9.46521700	5.95923700	0.00163200
C	-7.70892300	6.50335500	-0.42624000
C	-9.94469900	6.57250000	1.72276700
C	-10.68796800	6.60344900	-1.28608700
Si	9.46210900	5.92324900	0.00894100
C	10.70247200	6.57473200	-1.25823100
C	9.91509200	6.53067800	1.73930700
C	7.71043000	6.45972200	-0.44309600
Si	9.46401900	-5.95929000	0.00255700
C	9.94453400	-6.57220000	1.72352800
C	10.68563900	-6.60427800	-1.28584700
C	7.70724200	-6.50297200	-0.42389700
H	-2.71205000	3.47841000	-0.00226300
H	2.71187200	-3.47805400	-0.00076500
H	-4.77226500	-2.49753300	0.00011800
H	-4.79169100	2.52360700	-0.00175800
H	4.77218900	2.49780400	-0.00066500
H	4.79146400	-2.52330700	-0.00107000
H	-12.01051200	-2.47537300	-0.00223000
H	-12.00408200	2.51627400	-0.00458400
H	-14.49167000	-2.48282600	-0.00434800
H	-16.62442500	-1.22482300	-0.00677900
H	-16.62055000	1.27745600	-0.00807900
H	-14.48474500	2.52954600	-0.00691100

H	12.01070800	2.47499300	-0.00406900
H	12.00366200	-2.51665400	-0.00538600
H	14.49181900	2.48216000	-0.00719100
H	16.62443200	1.22391900	-0.01004600
H	16.62028000	-1.27836100	-0.01069500
H	14.48433500	-2.53021400	-0.00849600
H	0.81411500	4.53143200	-2.46961200
H	1.97594900	5.54791200	-1.57696700
H	2.16493300	3.77186000	-1.58121200
H	0.81599400	4.53384200	2.46397300
H	1.97691300	5.54961300	1.56934600
H	2.16611900	3.77346800	1.57519000
H	-1.57841600	5.79749400	-0.89502800
H	-1.57734700	5.79834900	0.89046500
H	-0.43709300	6.82619500	-0.00347300
H	-0.81458600	-4.53234400	-2.46743300
H	-1.97632500	-5.54832300	-1.57405000
H	-2.16527500	-3.77225200	-1.57924700
H	-0.81594400	-4.53226400	2.46616000
H	-1.97708200	-5.54832700	1.57210500
H	-2.16611200	-3.77219900	1.57715800
H	0.43685900	-6.82581100	-0.00026100
H	1.57809900	-5.79756900	-0.89246500
H	1.57722600	-5.79753400	0.89302800
H	-7.03960400	-6.06306300	0.62442000
H	-7.57554900	-7.55532300	-0.19521500
H	-7.24300900	-6.08077200	-1.14414800
H	-11.55777000	-6.24865300	-1.32957200
H	-10.48796500	-7.66895800	-1.45983100
H	-10.13043600	-6.19214700	-2.39374700
H	-10.12744200	-7.64569200	1.69222300
H	-9.56577900	-6.15740500	2.49927900
H	-11.19813000	-6.22290500	1.78970200
H	-7.41253600	6.13485900	-1.42145500
H	-7.62934900	7.60348400	-0.43107300
H	-6.98650100	6.11162500	0.30776800
H	-10.96080600	6.24338900	1.99395200
H	-9.92190800	7.67462200	1.76385800
H	-9.25070600	6.18869000	2.48773400
H	-10.43681100	6.23277000	-2.29291300
H	-11.71654900	6.28232800	-1.05490100
H	-10.67622500	7.70604600	-1.31660000
H	10.46810200	6.20570100	-2.26970100
H	10.68666600	7.67734600	-1.28635100
H	11.72869000	6.25726200	-1.01198100
H	9.21113800	6.14166000	2.49242200
H	9.88781100	7.63255500	1.78419000
H	10.92831800	6.20398200	2.02383500
H	7.62501300	7.55944700	-0.44208900
H	7.43159100	6.09602000	-1.44507500
H	6.97846000	6.05872200	0.27618600
H	9.25115600	-6.18801400	2.48886500
H	9.92150500	-7.67430400	1.76493700
H	10.96090500	-6.24326000	1.99393100
H	10.43392700	-6.23375200	-2.29259000
H	10.67346100	-7.70687900	-1.31607800

H	11.71450000	-6.28348600	-1.05545000
H	7.62728400	-7.60307500	-0.42825900
H	6.98546000	-6.11071400	0.31045700
H	7.41030200	-6.13475200	-1.41905000

7 Cyclic Voltammetry and Differential Pulse Voltammetry

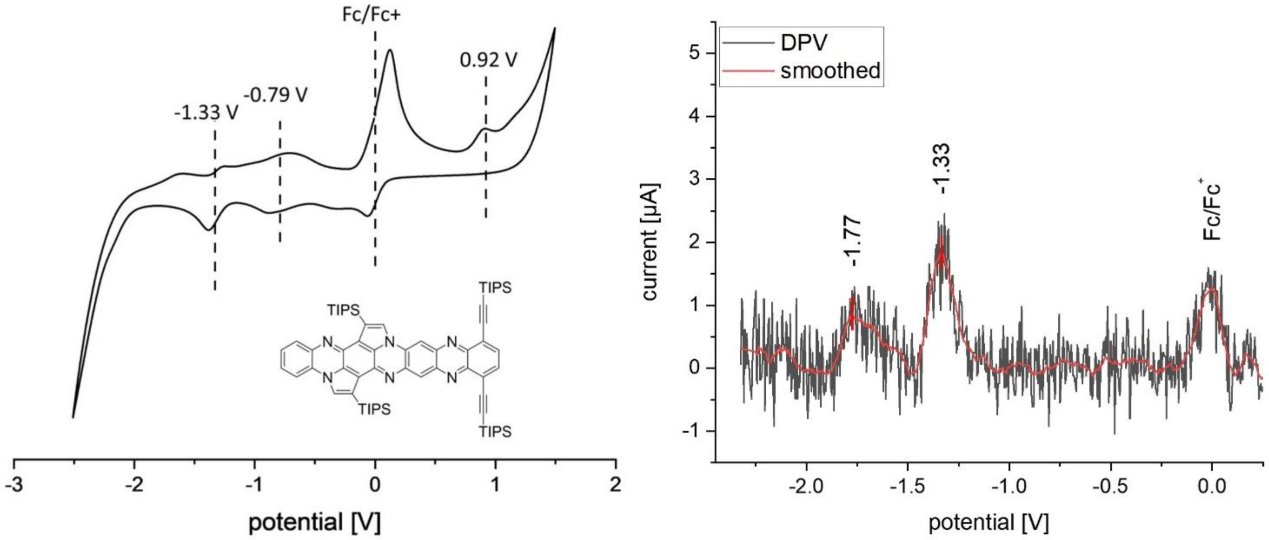

Figure 25. Left: CV spectrum of **2a** in DCM containing ferrocene as internal standard. Right: DPV spectrum of **2a** in DCM containing ferrocene as internal standard.

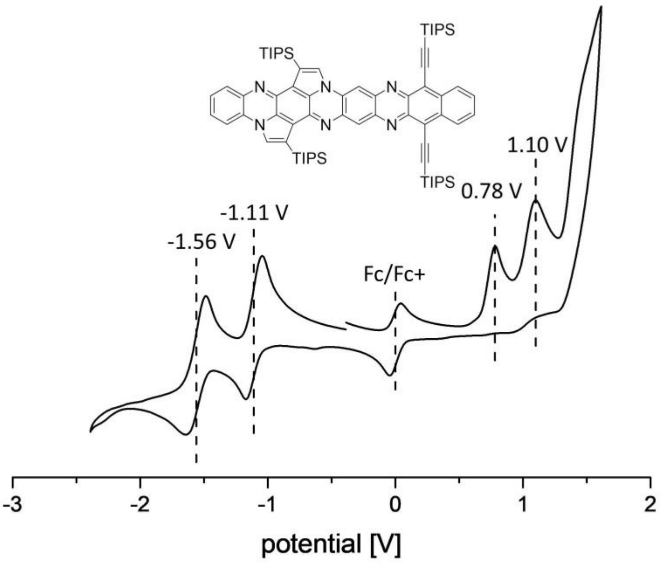

Figure 26. CV spectrum of **2b** in DCM containing ferrocene as internal standard.

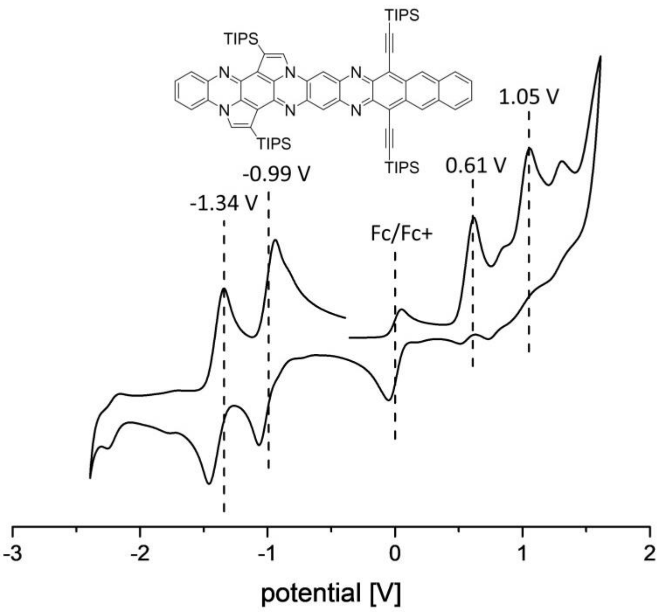

Figure 27. CV spectrum of **2c** in DCM containing ferrocene as internal standard.

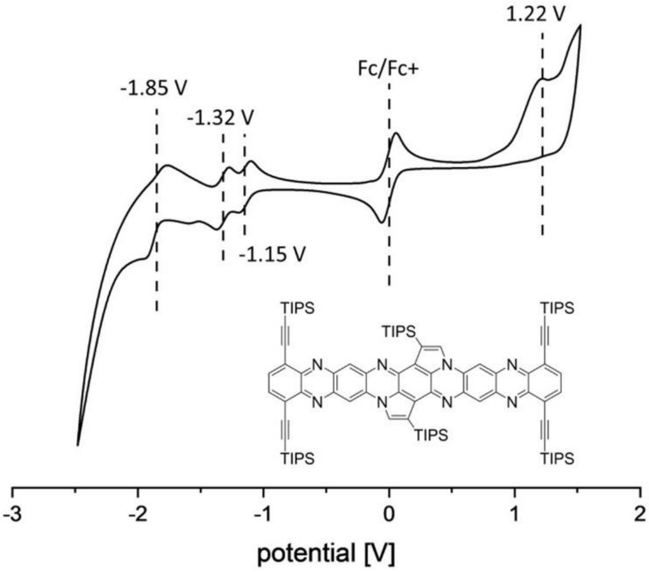

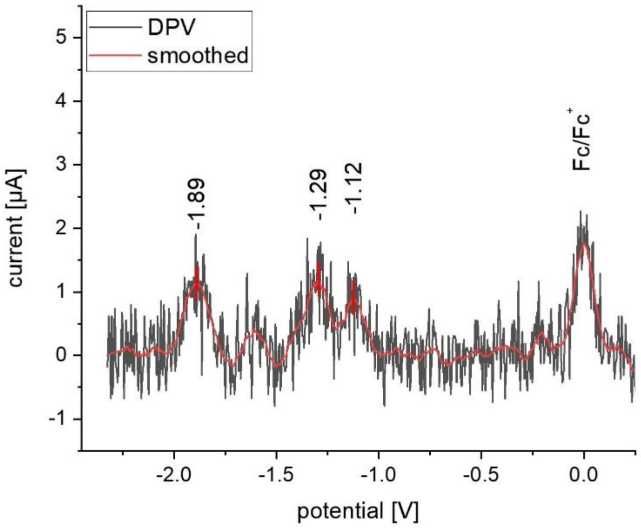

Figure 28. Left: CV spectrum of **3a** in DCM containing ferrocene as internal standard. Right: DPV spectrum of **3a** in DCM containing ferrocene as internal standard.

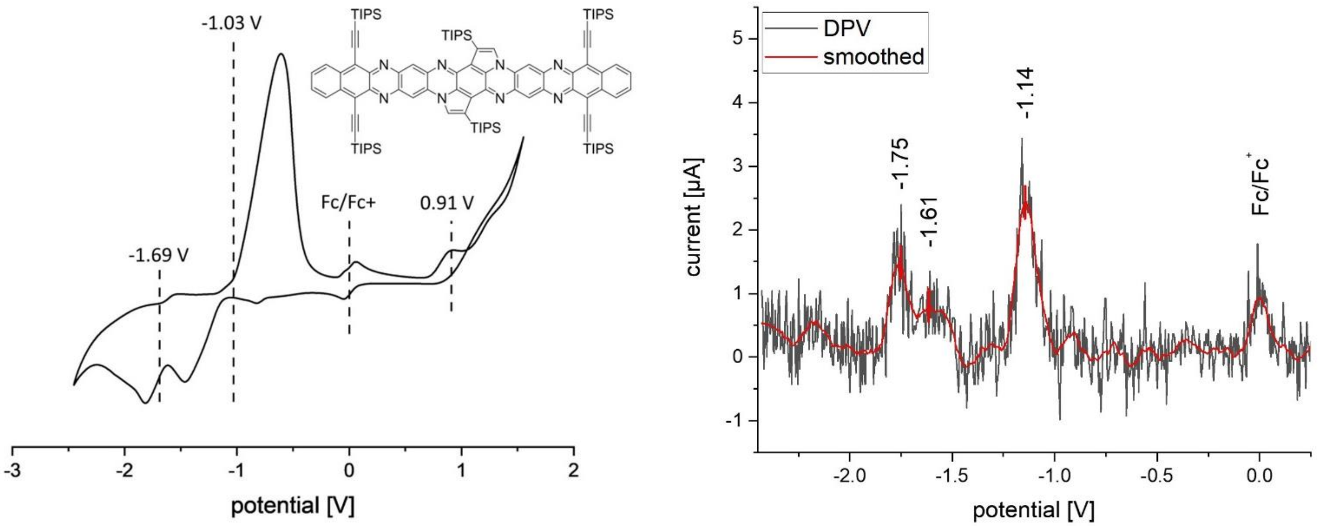

Figure 29. Left: CV spectrum of **3b** in DCM containing ferrocene as internal standard. Right: DPV spectrum of **3b** in DCM containing ferrocene as internal standard.

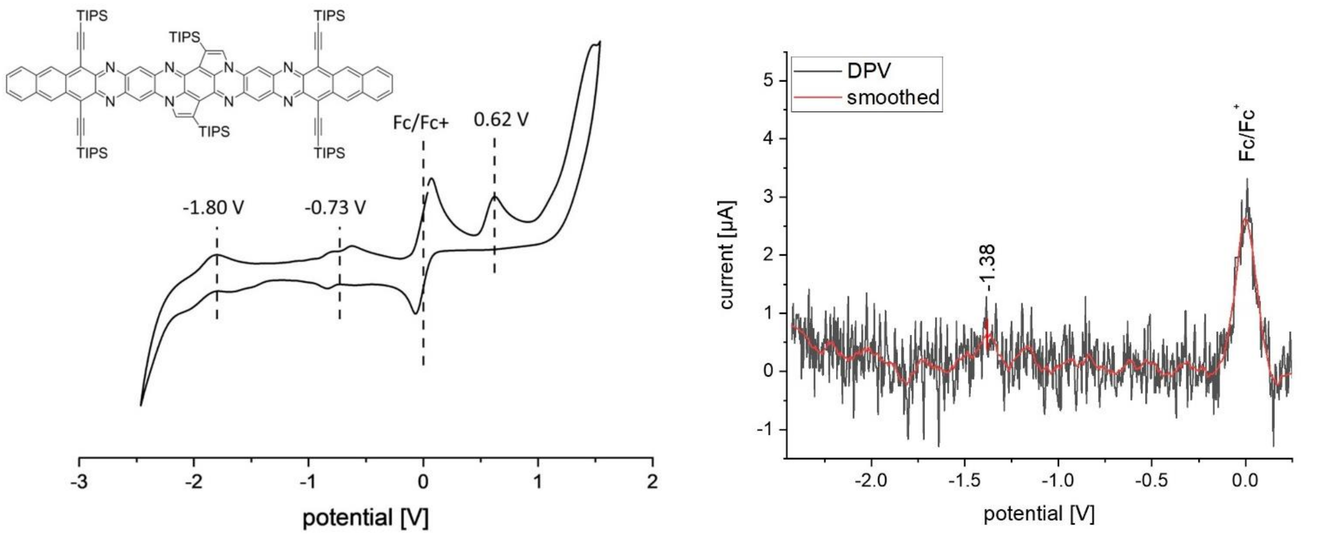

Figure 30. Left: CV spectrum of **3c** in DCM containing ferrocene as internal standard. Right: DPV spectrum of **3c** in DCM containing ferrocene as internal standard.

8 Device Manufacturing and Characterization

A sliced, highly doped silicon wafer with 100 nm thick thermally grown SiO_2 was cleaned by ultra-sonication in acetone, isopropanol and ethanol successively for 10 min. It was washed with water and dried in a stream of nitrogen. The wafer was placed in freshly produced Caro´s acid and heated to 100 °C for 20 min. After cleaning with water and drying, a 150 mM solution of $Al(NO_3)_3$ * 9 H_2O in ethanol was spin-coated (5000 rpm; 40 s) onto the substrate. Right after that the wafer was heated to 300 °C for 30 min. For the formation of the self-assembled monolayer, the substrate was placed in a 15.0 mM solution of 12-cyclohexyldodecylphosphonic acid (CDPA)[10] in isopropanol for 16 h. Then the substrate was cleaned with ultra-sonication in isopropanol for 10 min, rinsed with water and dried in a stream of nitrogen. The capacitance of the dielectric layer is amounted to 26.5 nF cm^{-2}.
Drop-cast films were prepared by dropping the prepared solution (**2b**: toluene, 0.5 mg/mL; **3b**: DCM, 0.5 mg/mL) onto the substrate and covering the wafer against unwanted wind flows. To form the electrodes, a 40 nm thick layer of silver was deposited through a shadow mask onto the organic layer in a vacuum evaporator at a pressure below 2 x 10^{-6} bar. Transistor characteristics were measured with a semiconductor characterization system (Keithley 4200-SCS) in a nitrogen filled glove box. The field effect mobilities were determined in the saturated regime using the equation $\mu = \left(\frac{\partial\,(I_{DS})^{1/2}}{\partial\,V_G}\right)^2 \frac{2L}{W\,C_i}$,

where I_{DS} is the source-drain current, W is the channel width, L the channel length, C_i is the capacitance per unit area of the gate dielectric layer, μ is the field effect mobility.

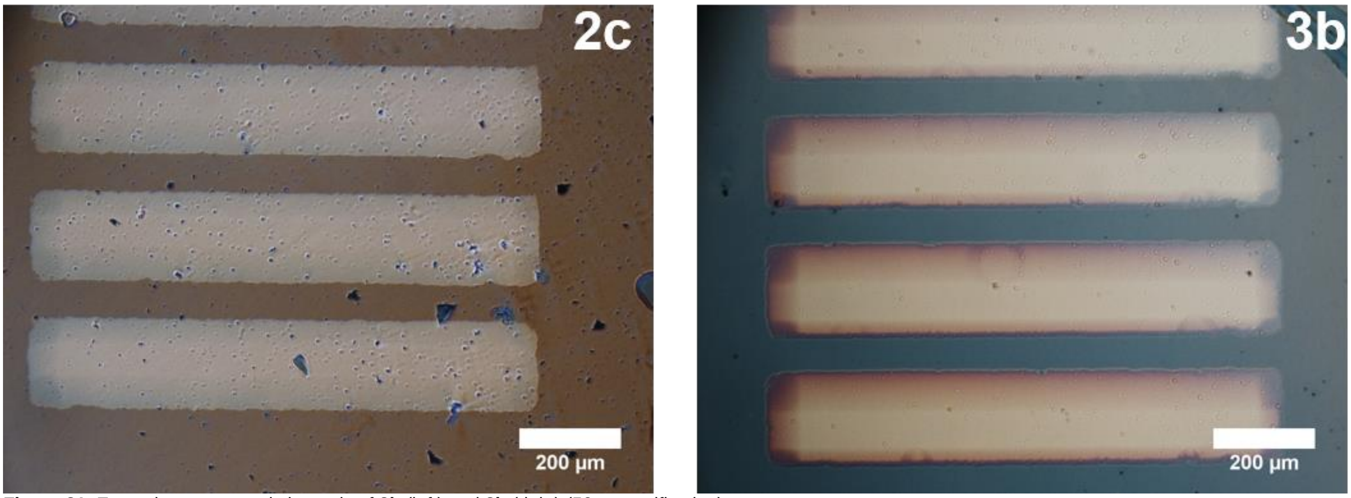

Figure 31. Exemplary measured channels of **2b** (left) and **3b** (right) (50x magnification)

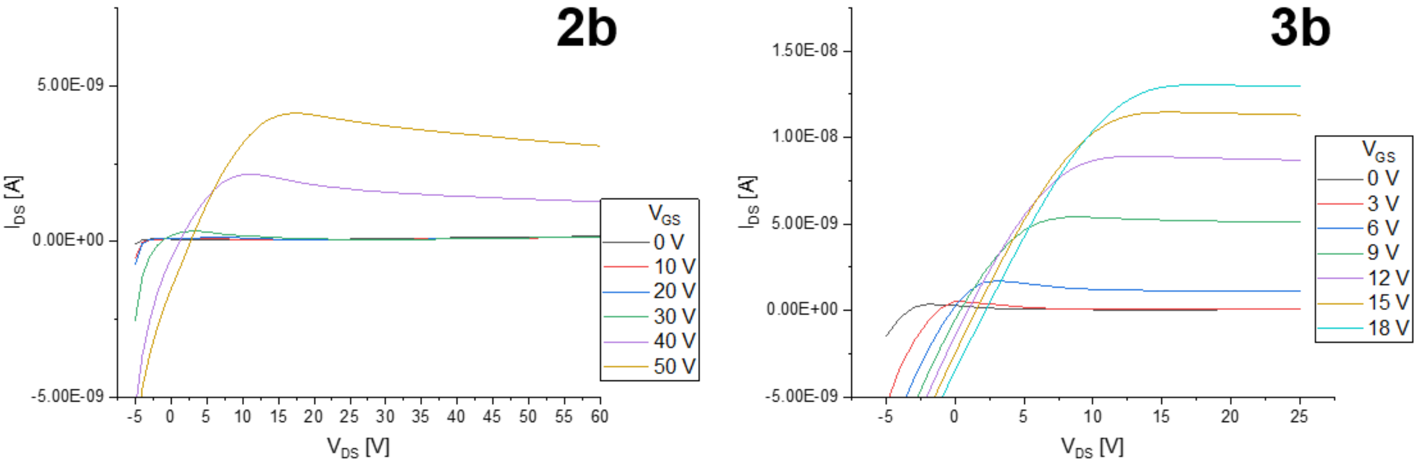

Figure 32. Output characteristics of **2b** and **3b** (right).

9 Stability Measurements

Stabilities of the compound were measured by time dependent absorption spectroscopy. The solutions of the compounds in DCM were irradiated with UV light (365nm) and white light under ambient conditions. In the figure below our LED panel is shown. The yellow LED were used for the measurement. All other LED were switched off.

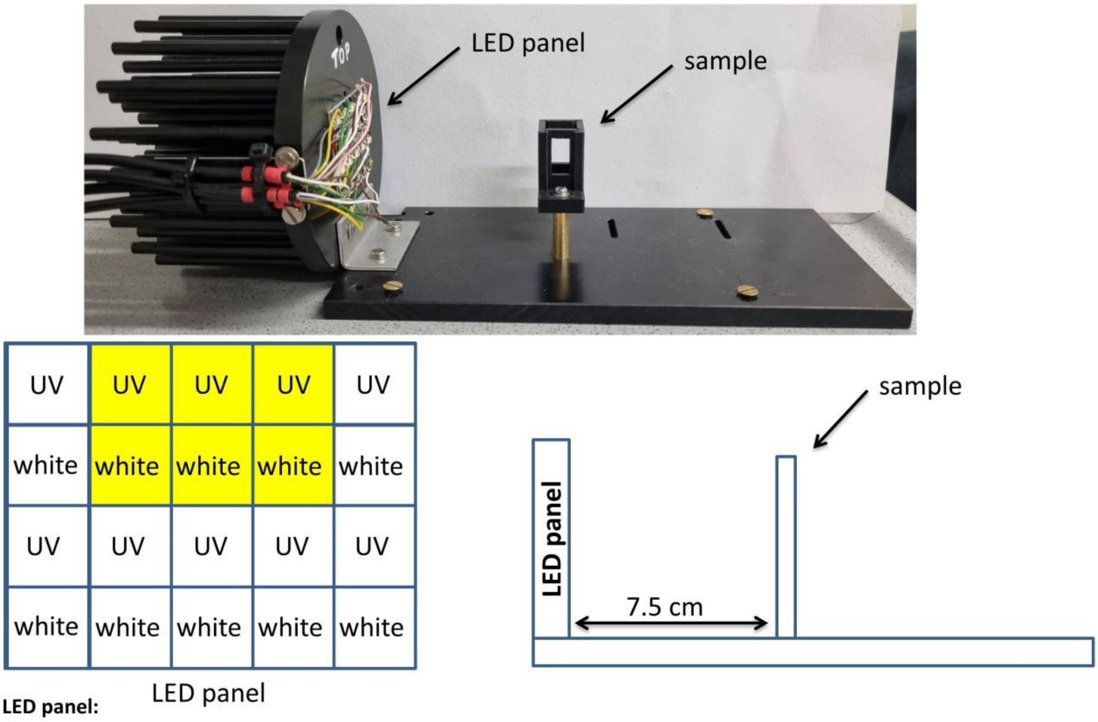

LED panel:
UV:
UV-LED: Nichia NCSU276C UV SMD-LED, 10x10mm Platine, 1050mW, 365nm

white:
white light LED: Cree XP-G2 S2 SMD-LED, 10x10mm Platine, 338lm, 6200K, CRI 70

Figure 33. Experimental setting for the irradiation experiments.

10 References

[1] G. R. Fulmer, A. J. M. Miller, N. H. Sherden, H. E. Gottlieb, A. Nudelman, B. M. Stoltz, J. E. Bercaw, K. I. Goldberg, *Organometallics* **2010**, *29*, 2176–2179.

[2] a) J. U. Engelhart, B. D. Lindner, O. Tverskoy, F. Rominger, U. H. F. Bunz *J. Org. Chem*. **2013**, *78*, 10832-10839; b) S. Miao, S. M. Brombosz, P. v. R. Schleyer, J. I. Wu, S. Barlow, S. R. Marder, K. I. Hardcastle, U. H. F. Bunz *J. Am. Chem. Soc.* **2008**, *130*, 7339-7344; c) A. L. Appleton, S. Miao, S. M. Brombosz, N. J. Berger, S. Barlow, S. R. Marder, B. M.Lawrence, K. I. Hardcastle, U. H. F. Bunz, *Org. Lett*. **2009**, *11*, 5222-5225.

[3] S. Maier, R. Heckershoff, N. Hippchen, K. Brödner, F. Rominger, J. Freudenberg, A. S. K. Hashmi, U.H. F. Bunz, *Chem. Eur. J*. **2022**, Accepted Author Manuscript.

[4] *Gaussian 16, Revision B.01,* M. J. Frisch, G. W. Trucks, H. B. Schlegel, G. E. Scuseria, M. A. Robb, J. R. Cheeseman, G. Scalmani, V. Barone, G. A. Petersson, H. Nakatsuji, X. Li, M. Caricato, A. V. Marenich, J. Bloino, B. G. Janesko, R. Gomperts, B. Mennucci, H. P. Hratchian, J. V. Ortiz, A. F. Izmaylov, J. L. Sonnenberg, D. Williams-Young, F. Ding, F. Lipparini, F. Egidi, J. Goings, B. Peng, A. Petrone, T. Henderson, D. Ranasinghe, V. G. Zakrzewski, J. Gao, N. Rega, G. Zheng, W. Liang, M. Hada, M. Ehara, K. Toyota, R. Fukuda, J. Hasegawa, M. Ishida, T. Nakajima, Y. Honda, O. Kitao, H. Nakai, T. Vreven, K. Throssell, J. A. Montgomery, Jr., J. E. Peralta, F. Ogliaro, M. J. Bearpark, J. J. Heyd, E. N. Brothers, K. N. Kudin, V. N. Staroverov, T. A. Keith, R. Kobayashi, J. Normand, K. Raghavachari, A. P. Rendell, J. C. Burant, S. S. Iyengar, J. Tomasi, M. Cossi, J. M. Millam, M. Klene, C. Adamo, R. Cammi, J. W. Ochterski, R. L. Martin, K. Morokuma, O. Farkas, J. B. Foresman, and D. J. Fox, Gaussian, Inc., Wallingford CT, **2016**.

[5] a) G. te Velde, F.M. Bickelhaupt, E.J. Baerends, C. Fonseca Guerra, S.J.A. van Gisbergen, J.G. Snijders, T. Ziegler, Journal of Computational Chemistry, 2001 22, 931 ; b) ADF 2022.1, SCM, Theoretical Chemistry, Vrije Universiteit, Amsterdam, The Netherlands,

[6] V. Stehr, J. Pfister, R. F. Fink, B. Engels, C. Deibel, *Physical Review B* **2011**, *83*, 155208.

[7] R. A. Marcus, *Reviews of Modern Physics* **1993**, *65*, 599-610.
[8] a) S.-H. Wen, A. Li, J. Song, W.-Q. Deng, K.-L. Han, W. A. Goddard, *J. Phys. Chem. B* **2009**, *113*, 8813-8819; b) A. N. Sokolov, S. Atahan-Evrenk, R. Mondal, H. B. Akkerman, R. S. Sánchez Carrera, S. Granados-Focil, J. Schrier, S. C. B. Mannsfeld, A. P. Zoombelt, Z. Bao, A. Aspuru-Guzik, *Nature Communications* **2011**, *2*, 437.
[9] a) S.-H. Wen, A. Li, J. Song, W.-Q. Deng, K.-L. Han, W. A. Goddard, *J. Phys. Chem. B* **2009**, *113*, 8813-8819; M. Chu, J.-X. Fan, S. Yang, D. Liu, C. F. Ng, H. Dong, A.-M. Ren, Q. Miao, *Adv. Mater.* **2018**, *30*, 1803467
[10] D. Liu, Z. He, Y. Su, Y. Diao, S.C.B. Mannsfeld, Z. Bao, J. Xu, Q. Miao. Adv. Mater. 2014, 26: 7190-7196
